# Probabilistic ecological risk assessment for deep‐sea mining: A Bayesian network for Chatham Rise, Pacific Ocean

**DOI:** 10.1002/eap.3064

**Published:** 2024-11-25

**Authors:** Laura Kaikkonen, Malcolm R. Clark, Daniel Leduc, Scott D. Nodder, Ashley A. Rowden, David A. Bowden, Jennifer Beaumont, Vonda Cummings

**Affiliations:** ^1^ National Institute of Water and Atmospheric Research Wellington New Zealand; ^2^ Ecosystems and Environment Research Programme University of Helsinki Helsinki Finland; ^3^ Finnish Environment Institute Helsinki Finland; ^4^ Victoria University of Wellington Wellington New Zealand

**Keywords:** Bayesian networks, benthic fauna, deep‐sea mining, participatory modeling, quantitative risk assessment, seabed disturbance

## Abstract

Increasing interest in seabed resource use in the ocean is introducing new pressures on deep‐sea environments, the ecological impacts of which need to be evaluated carefully. The complexity of these ecosystems and the lack of comprehensive data pose significant challenges to predicting potential impacts. In this study, we demonstrate the use of Bayesian networks (BNs) as a modeling framework to address these challenges and enhance the development of robust quantitative predictions concerning the effects of human activities on deep‐seafloor ecosystems. The approach consists of iterative model building with experts, and quantitative probability estimates of the relative decrease in abundance of different functional groups of benthos following seabed mining. The model is then used to evaluate two alternative seabed mining scenarios to identify the major sources of uncertainty associated with the mining impacts. By establishing causal connections between the pressures associated with potential mining activities and various components of the benthic ecosystem, our model offers an improved comprehension of potential impacts on the seafloor environment. We illustrate this approach using the example of potential phosphorite nodule mining on the Chatham Rise, offshore Aotearoa/New Zealand, SW Pacific Ocean, and examine ways to incorporate knowledge from both empirical data and expert assessments into quantitative risk assessments. We further discuss how ecological risk assessments can be constructed to better inform decision‐making, using metrics relevant to both ecology and policy. The findings from this study highlight the valuable insights that BNs can provide in evaluating the potential impacts of human activities. However, further research and data collection are crucial for refining and ground truthing these models and improving our understanding of the long‐term consequences of deep‐sea mining and other anthropogenic activities on marine ecosystems. By leveraging such tools, policymakers, researchers, and stakeholders can work together toward human activities in the deep sea that minimize ecological harm and ensure the conservation of these environments.

## INTRODUCTION

Interest in seabed mining, deep‐sea fishing, and oil and gas exploration is increasing in the deep sea (Jouffray et al., 2020; Ramirez‐Llodra et al., 2011). In order to effectively manage these human activities, predicting their impacts on deep‐sea environments and ecosystems prior to resource consent approval is crucial. However, the complexity of deep‐sea ecosystems and the lack of comprehensive data often make predicting impacts challenging (Smith et al., 2020). It is therefore important to conduct robust environmental risk assessments (ERAs) that consider the potential risks and uncertainties associated with activities that can impact deep‐sea habitats and communities.

Significant mineral resources have been identified in various parts of the global ocean, including areas of the Pacific, Atlantic, and Indian oceans (Ellis et al., 2017; Miller et al., 2018). Seabed mining activities are still in the exploration and development phase and no commercial mining operations have yet taken place, but concerns have been raised over their potential to harm deep‐sea ecosystems (van Dover et al., 2017). Deep‐sea mining is expected to have an impact on all levels of marine ecosystems, from the water column to the seafloor (Drazen et al., 2020; Miljutin et al., 2011; Orcutt et al., 2020). Many studies have examined the potential environmental consequences of mining using field studies (reviewed by Jones et al. (2017)), laboratory experiments (Brown & Hauton, 2018; Gillard et al., 2019; Mobilia et al., 2021), and modeling (Purkiani et al., 2021; Stratmann et al., 2018). However, despite the valuable insights provided by these studies, there is only a partial understanding of the environmental impacts of deep‐sea mining. Current knowledge gaps include uncertainties about the scale and duration of the effects, the potential for cumulative impacts over time, and the extent and speed of ecosystem recovery following disturbance (Amon et al., 2022). Furthermore, it is unclear to what extent the disturbance studies conducted in a small area or in a laboratory can be scaled up to industrial mining operations (Clark et al., 2020). Limited baseline data and the difficulty of access to some of the remote areas where deep‐sea mining is proposed make it challenging to accurately assess the environmental risks associated with such mining activities (Smith et al., 2020).

ERAs are an important tool to help environmental managers evaluate the risks associated with mining operations. In the deep sea, ERAs can be particularly useful to support decision‐making, due to limitations of baseline data and of information on ecosystem responses to external disturbances. However, most current ERAs estimate risk based upon the vulnerability of the environment through semiquantitative scoring (Boschen‐Rose et al., 2021; Washburn et al., 2019), offering an overview of the risks without quantitative estimates of the actual ecosystem impacts. To account for the uncertainties related to such lack of data, probabilistic modeling has shown its increasing value when used in ERAs (Kaikkonen, Helle, et al., 2021; Kaikkonen, Parviainen, et al., 2021).

Bayesian networks (BNs) are graphical probabilistic models that provide an alternative to commonly used scoring procedures in ERAs (Kelly et al., 2013; Pearl, 2010). In a risk assessment context, BNs illustrate the modeled system as a network of causal influences. BNs are composed of a directed acyclic graph (the structure of the network) with quantitative connections between the variables (or nodes). The strength of each connection between variables is described through conditional probabilities (Pearl, 1986), thus representing a joint probability distribution over a set of variables. The dependencies between variables propagate through the network and influence the probabilities of other nodes and may be updated as new information about the nodes becomes available. This facet of the model enables the integration of new data or evidence in the model, and the network can be queried under different scenarios to calculate the posterior probability of all other nodes within the BN (Kelly et al., 2013; Pearl, 2010).

Unlike traditional scoring procedures, BNs allow for the estimation of not only the most likely outcome but also the uncertainty associated with the estimates by providing a probability distribution over all the possible values of each variable (Fenton & Neil, 2012; Nielsen & Jensen, 2009). BNs can synthesize outcomes of multiple scenarios and accommodate inputs from multiple sources, including simulations, empirical data, and expert knowledge (e.g., Bulmer et al., 2022; Wade et al., 2021), making them well‐suited for data‐poor cases. Additionally, given their modular structure, BNs can support integrative modeling combining different submodels, such as management decision networks (Marcot & Penman, 2019).

In this paper, we apply BNs in a case study focused on potential phosphate nodule mining on the Chatham Rise, offshore Aotearoa/New Zealand, Southwest Pacific Ocean. Drawing on a combination of field observations, laboratory experiments, and expert knowledge, we estimate the likelihoods of impacts on benthic fauna under a high disturbance and an intermediate disturbance seabed mining scenarios.

## MATERIALS AND METHODS

### Chatham rise phosphate nodule mining case study

#### Background

**FIGURE 1:**
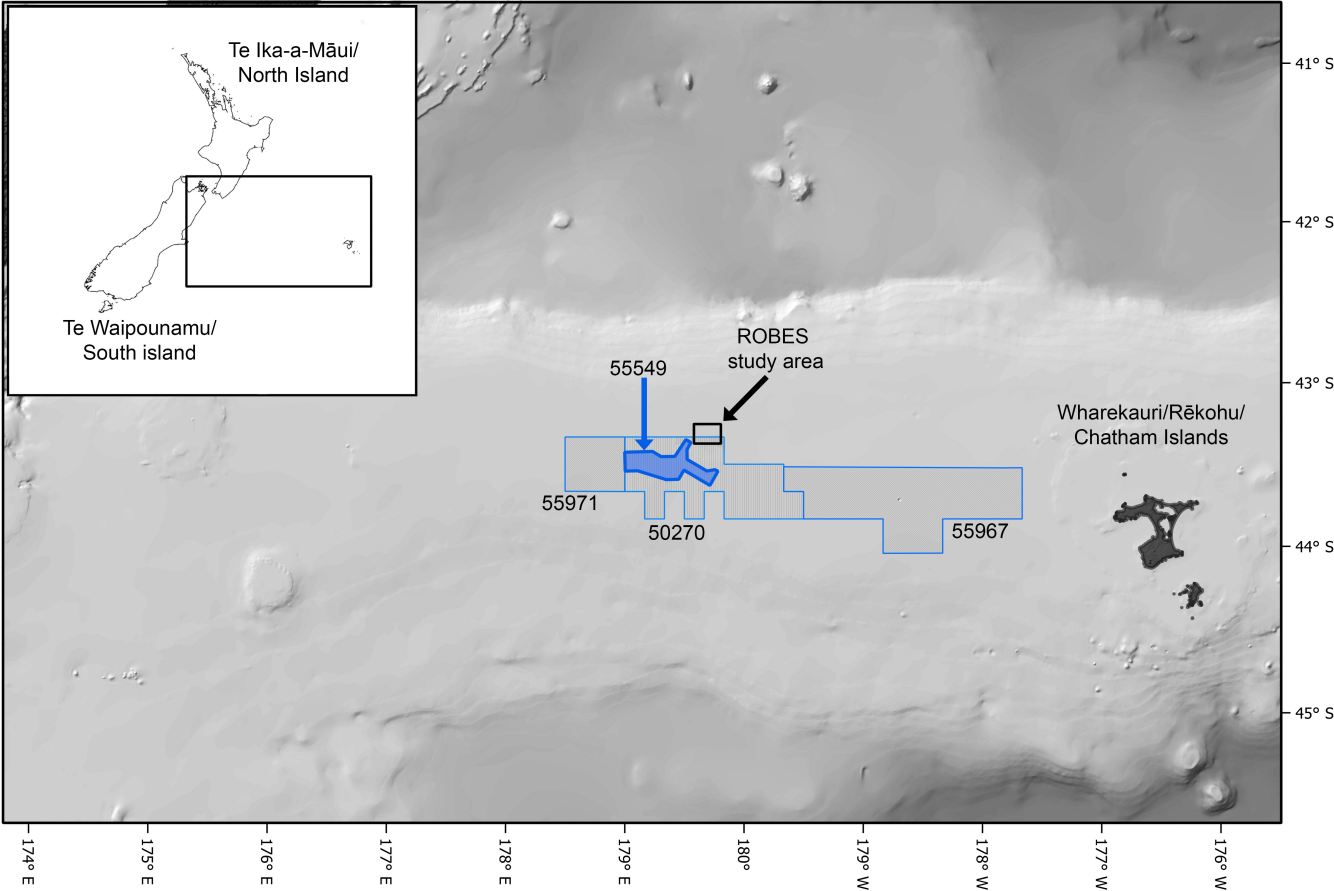
Map of the study area on the Chatham Rise, offshore of New Zealand. The numbered polygons denote the CRP Minerals Mining Permit (55549) and previous Mineral Prospecting Permit areas.

In 2013, a New Zealand company, Chatham Rock Phosphate (CRP), applied for and was granted a Minerals Mining Permit by the New Zealand Government for phosphate nodule extraction from the seafloor on the Chatham Rise, located in the central eastern region of New Zealand's 200 nautical mile Exclusive Economic Zone (EEZ) (Figure 1). The depth of the crest of the Chatham Rise is 200–500 m and its flanks deepen to more than 2000 m to the north and south (Nodder et al., 2012). The area is characterized by high primary productivity, with dynamic oceanographic conditions characterized by variable currents and interweaving water masses associated with the Subtropical Frontal Zone (Collins et al., 2023; Murphy et al., 2001; Safi et al., 2023). The sediments covering the crest are predominantly organic‐rich, glauconitic muddy sands and sandy muds, with phosphorite nodules and hardgrounds present on top of and within the sediment (Cullen, 1987; Nelson et al., 2022; Norris, 1964). The seafloor communities in the area are characterized by a wide range of invertebrate species, many of which are infaunal, although some species occupy habitat niches either on top of the sediment (epifauna) or just above the seafloor within the hyperbenthos (Compton et al., 2013). Corals and other sessile epifaunal organisms, such as sponges, also live attached to hard substrates such as phosphorite nodules or rock outcrops (Dawson, 1984; Leduc et al., 2015; Nodder et al., 2012; Rowden et al., 2014).

The proposed mining operation by CRP was to extract phosphorite nodules from the seafloor using a trailing suction drag‐head and to mechanically process the nodules on board the mining support vessel. Nodules larger than 2 mm in diameter would be separated from other sediment material and the waste would then be discharged close to the seafloor via a discharge pipe (CRP, 2014). The mining would be carried out over separate mining blocks, each covering an area of 5 km × 2 km and taking approximately 14 weeks to complete mining operations. However, in 2015, the marine consent application to carry out the mining of phosphorite nodules was denied, due in part to uncertainty surrounding the potentially adverse effects on biological communities, including impacts caused by suspended and deposited sediment (NZ EPA, 2015).

In order to address the scientific uncertainties related to impacts from seabed sediment disturbance, the “Resilience of deep‐sea benthic communities to the effects of sedimentation” (ROBES) program gathered information on various environmental factors and benthic fauna. The program consisted of both field and laboratory simulations to characterize the benthic effects of an artificial physical seabed disturbance and associated sediment plume on the Chatham Rise crest in the vicinity of the proposed CRP phosphorite mining area (Figure 1). The experimental seabed disturbances, although not equivalent to actual seabed mining, were anticipated to provide important insights into the impacts of deep‐sea mining and other significant benthic disturbances such as bottom trawling (Clark et al., 2018).

#### Data

The data used in this study originate from the field and laboratory measurements collected through the ROBES program in 2018–2020 (see details of the data used in Appendix S1: Table S1). The fieldwork took place on the northern edge of the Chatham Rise crest at depths of 400–500 m (Figure 1). The study area was surveyed in 2018 and 2019, then a targeted area was artificially disturbed using a mechanical disturber (a modified agricultural plow and a harrow mat) and sampled immediately after the disturbance in 2019 and one year later in June 2020 (see details in Clark, Nodder, Leduc, Eager, et al., 2021; Clark, Nodder, Leduc, Hale, et al., 2021; Leduc et al., 2024). A diverse range of data was collected to characterize the site, encompassing oceanographic (Acoustic Doppler current profiler [ADCP], ocean glider, moorings, conductivity, temperature, and depth [CTD], acoustics) and nearbed sediment conditions (benthic landers, sediment trap moorings, multicorer, onboard sediment experiments), and benthic communities. Environmental baseline conditions were determined using year‐long ADCP moorings and glider and CTD deployments during the ROBES voyages, followed by modeling and analysis of satellite remote sensing data (Collins et al., 2023). Information on baseline nearbed particle and organic carbon fluxes were derived from biweekly and daily sampling using moored and lander sediment traps, respectively. Macroinfauna and meiofauna samples were collected before and after the disturbance using a multicorer with replicate samples from sites that were directly impacted by the mechanical disturber and from near‐field areas that were expected to be subject to sedimentation (see Clark et al., 2019; Clark, Nodder, Leduc, Eager, et al., 2021; Clark, Nodder, Leduc, Hale, et al., 2021; Leduc et al., 2024). In addition to the field sampling, live sponges and corals were transported back to the laboratory to assess their response to different concentrations and frequencies of suspended sediment over time (for details, see Mobilia et al., 2021, 2023).

### BN modeling

#### Model development and variable selection

A conceptual influence diagram synthesizing the impacts of deep‐sea mining on the Chatham Rise was developed in a series of workshops with experts (Appendix S1: Table S3). Given the complexity of the Chatham Rise ecosystem, we focus only on benthic ecosystem impacts associated with seabed mining. Due to lack of empirical data from ROBES and other regional experts, we did not consider impacts from noise and vibrations associated with the mining activities in our assessment nor the environmental effects on certain components of the marine foodweb, such as bacteria, marine mammals, and seabirds. We note that in a real‐world application of the proposed ERA approach, inclusion of these ecosystem components is essential.

The causal network resulting from the expert elicitation was developed into a BN model in an iterative manner in expert workshops by selecting key variables to evaluate and defining relevant variable states (for details of the model building methodology, see Appendix S1; Kaikkonen, Helle, et al., 2021). To facilitate model quantification and ensure model parsimony, the number of parent nodes was limited whenever possible (Chen & Pollino, 2012).

Discrete variable states were defined based on data, literature, and expert views (Table 1, full references in Appendix S1: Table S4). Variable states were chosen to represent likely variations in the variables of interest in the context of seabed disturbance on the Chatham Rise. For variables that describe the implementation of the potential mining activity (hereafter “operational variables”), variable states were drawn from the environmental consent application prepared by CRP (2014). The states of the physicochemical variables, describing the environmental conditions and associated changes from mining, were defined by experts based on field observations, expert knowledge, and primary literature. For variables that directly affect benthic fauna, such as suspended sediment concentration (SSC), the states were set to reflect biologically relevant thresholds whenever possible (e.g., Hewitt & Lohrer, 2013; Mobilia et al., 2021, 2023). As a result, the states do not always follow a continuous scale nor cover all possible values of a variable but were selected to represent likely outcomes from different disturbance events (see Appendix S1: Table S4 for rationale for variable discretization).

**TABLE 1:** Physical and environmental model variables and the methods used to discretize and parameterize the variables used in the Bayesian network modeling.

Variable name	Description	Variable type	Variable states	Discretization based on	Parameterization based on
Depth of extracted sediment	Depth of sediment extracted by the mining tool	Decision variable	<10 cm/10–30 cm/>30 cm	Literature, expert opinion	Not applicable
Processing return technique	Depth of processing return water and sediment	Decision variable	10 m from seafloor/at the seafloor	Literature	Not applicable
Mining intensity	Proportion of area mined within a discrete mining block	Decision variable	50%/75%/100%	Expert opinion	Not applicable
Distance from the mining block	Distance from the mining block	Decision variable	Inside mining block/near‐field/far‐field	Literature, expert opinion	Not applicable (decision variable)
Volume of extracted sediment	Volume of sediment removed by a mining operation tool (as the initial removal)	Random variable	Low/medium/high	Literature, expert opinion	Literature, expert assessment
Particle size composition of the extracted sediment	Proportion of fine and coarser particles of the extracted substrate and in the composite sediment plume	Random variable	Mostly silt and clay (fine)/mix of fine and coarse particles/mostly coarse (sand and gravel)	Data, expert opinion	Field surveys
Sediment contaminants	Concentration of potentially harmful substances in the sediment and the mineral material to be extracted	Random variable	Low/medium/high	Literature, expert opinion	Literature, expert assessment
Nodule removal	Proportion of phosphorite nodules removed from a discrete mining block	Decision variable	Yes/no	Not applicable	Not applicable (decision variable)
Suspended sediment	Total suspended solids concentration near the seafloor resulting both from the processing return and mining tool operation	Random variable	Low: <10 mg L^−1^/moderate: 11–50 mg L^−1^/high: >100 mg L^−1^	Data, literature	Expert assessment informed by literature
Sediment deposition	Depth of sediment deposited from the composite suspended sediment plume	Random variable	Low: <1–2 cm/moderate: 2–5 cm/high: 10‐25 cm	Data, literature	Field measurements and expert assessment
Contaminant release	Release of contaminants from the sediment plume to the seabed water column	Random variable	Nonsignificant/significant release of contaminants	Data, literature	Expert assessment
Changes in sediment characteristics	Measure of changes in the sediment environment affecting habitat quality for benthic organisms	Random variable	Minor to no changes/significant changes	Data, expert opinion	Field measurements and expert assessment

*Note*: Random variables refer to variables with an associated probability distribution, whereas decision variables describe processes that are assumed to be controlled by the party responsible for the extraction activity and are thus nonrandom. Full rationale supporting the variable states and parameterization with references are presented in Appendix S1.

As the number of species found in the study area is too high to assess the impacts on each species or taxon separately, we reduced this complexity by grouping organisms into functional groups (Bremner, 2008). The functional groups were assigned based on traits that have been shown to influence the organisms' response to seafloor disturbance and recovery potential (e.g., body size, feeding habit, position in sediment, and mobility; Hewitt et al., 2018), using previously created groupings for the Chatham Rise as a starting point (Lundquist et al., 2018). These trait‐based groups encompassed a range of faunal groups across meiofauna, macroinfauna, epibenthos, and hyperbenthos (Table 2). Throughout the paper, we use the terms infauna (including meio‐ and macroinfauna), sessile benthos (including sessile invertebrate megafauna), and mobile benthos (describing mobile invertebrate megafauna) to further combine these functional groups. Examples and explanations of the faunal groups are given in Table 2.

**TABLE 2:** Biological model variables describing the benthic faunal functional groups and the methods used to discretize and parameterize the variables used in the Bayesian network modeling.

Variable name	Example taxa	Variable type	Variable states	Discretization method	Parameterization method
Sessile encrusting suspension feeders	Encrusting bryozoans, corals	Random variable	% decrease in abundance	Expert opinion	Expert assessment
Sessile encrusting filter feeders	Encrusting sponges	Random variable	% decrease in abundance	Expert opinion	Expert assessment
Sessile erect suspension feeders	Branching bryozoans, crinoids and corals (stony and octocorals)	Random variable	% decrease in abundance	Expert opinion	Laboratory experiment and expert assessment
Sessile erect filter feeders	Erect sponges	Random variable	% decrease in abundance	Expert opinion	Laboratory experiment and expert assessment
Soft‐bodied erect suspension and filter feeders	Small soft‐bodied hydroids, ascidians, small bryozoans	Random variable	% decrease in abundance	Expert opinion	Expert assessment
Deep meiofauna	Mainly nematodes	Random variable	% decrease in abundance	Expert opinion	Field measurements and expert assessment
Surface meiofauna	Mixed community of meiofauna	Random variable	% decrease in abundance	Expert opinion	Field measurements and expert assessment
Small sessile macroinfauna	Paraonid and capitellid polychaetes, small bivalves	Random variable	% decrease in abundance	Expert opinion	Field measurements and expert assessment
Small mobile macroinfauna	Mobile deposit feeders and small scavengers (amphipods, small crustaceans)	Random variable	% decrease in abundance	Expert opinion	Field measurements and expert assessment
Large sessile macroinfauna	Tube‐forming polychaetes, tube‐building amphipods, large bivalves	Random variable	% decrease in abundance	Expert opinion	Field measurements and expert assessment
Large mobile macroinfauna	Large burrowing polychaetes, some arthropods, large bivalves, asteroids	Random variable	% decrease in abundance	Expert opinion	Field measurements and expert assessment
Mobile deposit feeding or grazing epibenthos	Mobile deposit feeders, surface dwelling species, for example, spatangoid echinoids, holothurians, ophiuroids, gastropods	Random variable	% decrease in abundance	Expert opinion	Expert assessment
Mobile predatory or scavenging epibenthos	Mobile surface crawling predators & scavengers, for example, squat lobsters, crab, scampi, gastropods	Random variable	% decrease in abundance	Expert opinion	Expert assessment
Predatory or Scavenging hyperbenthos	Small swimming crustaceans (mysids, amphipods)	Random variable	% decrease in abundance	Expert opinion	Expert assessment
Grazing or deposit‐feeding hyperbenthos	Benthopelagic holothurians, benthopelagic gastropods, others	Random variable	% decrease in abundance	Expert opinion	Expert assessment

*Note*: Random variables refer to variables with an associated probability distribution. Data collection methods and full rationale supporting the variable states and parameterization are presented in Appendix S1: Tables S1 and S4.

#### Model parameterization

Within a BN, the magnitudes of impacts are illustrated through conditional dependencies. The probabilities of each value of the “child” node, conditioned on every possible combination of values of the “parent” nodes, can be drawn from data, expert opinion, or a combination of these two inputs (Barber 2012). Conditional probabilities (summarized in conditional probability tables, CPTs) were derived from a combination of data and expert assessment (Tables 1 and 2, details on the method below and in Appendix S1).

In order to generalize the impacts of stressors on the different functional groups and to reduce the elicitation burden on experts, we applied an interpolation method (Barons et al., 2022) to derive missing probability distributions. The method assumes that the child node can be estimated through a beta distribution and requires the user to identify both a “best case” and a “worst case” distribution for the child node. In this context, “best” and “worst” pertain to distributions where the parents affecting the node are at their highest and lowest points for the child, respectively. The probability distributions for all other combinations of parent variables are then inferred by interpolating between these extremes. This procedure is accomplished using a series of weights assigned to the parent states, which are employed to interpolate between the parameters of a beta distribution.

Where data on the impacts were available, empirical data were used as the starting point, which corresponded to the best case scenario, and experts were then asked to estimate: (1) the relative importance of each of the stressors on each functional group, and (2) the probability distribution for the relative decrease in abundance (compared to pre‐mining abundance) of each functional group under the worst case scenario (i.e., each stressor at the maximum state). The interpolation method can be applied to variables with ranked ordinal states (e.g., low to high). For nodes where such ordinal ranking was not appropriate (e.g., sediment type), we used the Application for Conditional probability Elicitation (ACE; Hassall et al., 2019) to initialize the CPTs based on a small number of simple questions that capture the overall shape of a conditional probability distribution. The application has a visual aid and allows to easily refine the shape of the distribution, which was undertaken together with the experts (see Appendix S1). All CPTs, as well as a more detailed description of the elicitation protocol, are available at github.com/lkaikkonen/CR-ERA, including comments on the rationale underlying the probability distributions for each response variable.

All CPTs were reviewed with the experts and adjusted when deemed necessary to ensure consistency in the estimated impacts with respect to expected differences between the different faunal groups. In addition to the probability estimates, experts evaluated their confidence in the estimates for each of the CPTs. The resulting CPTs were incorporated in the BN model created in R software. The modeling was conducted using R 3.6.3, with the R package *bnlearn* (Scutari, 2009). Full details of the model with the R scripts and the CPTs are available at github.com/lkaikkonen/CR-ERA.

#### Modeling framework and model structure

**FIGURE 2:**
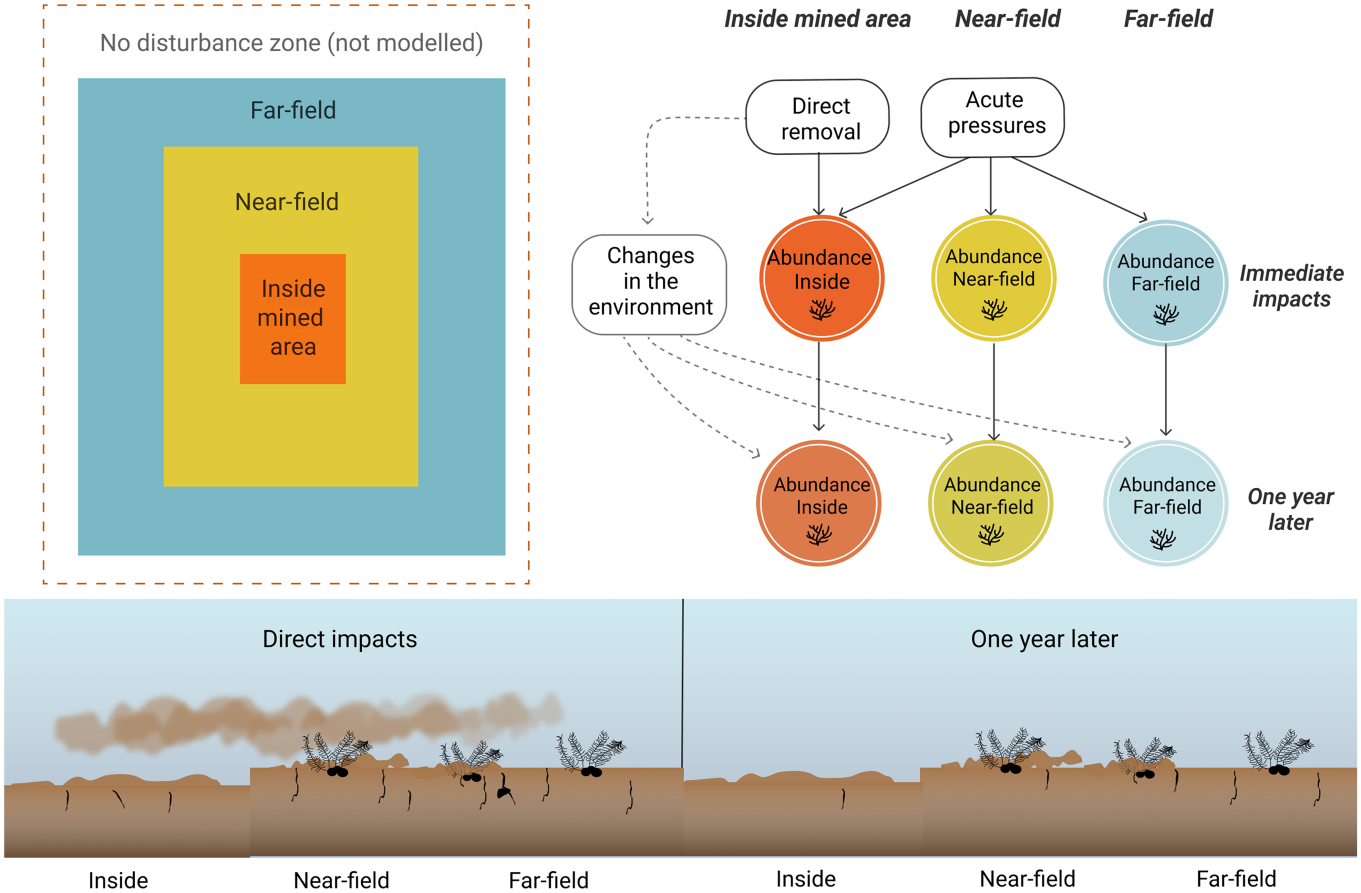
General modeling framework for impacts of seabed mining‐related disturbance in time and space for any given functional group in the Bayesian network model. By defining the effects for the separate time steps, the impacts may be assessed jointly or separately for each discrete area (depicted by the squares, upper left panel). The lower panel illustrates the spatial and temporal distribution of the pressures. Immediate impacts consist of direct extraction of sediment and nodules within the mining block, and elevated suspended sediment concentrations and redeposition of sediment in all areas resulting from the mechanical disturbance of the sediment and sediment discharge after nodules have been removed. Impacts after one year are a result of the altered sedimentary environment which affects the recovery potential of benthic organisms. The magnitude and likelihood of all these changes are variable and included in the probability assessments (see text for details). Image by Laura Kaikkonen, with all icons either created by Laura Kaikkonen or openly available from Iconscout under a license that allows reuse.

We consider three spatial domains in the model: the area inside the mining block that is mined (inside), areas immediately adjacent to the mining block that are not mined (near‐field), and areas further from the mining area that are still expected to be within the zone impacted by the mining activities (far‐field) (Figure 2). Unimpacted areas beyond far‐field are not included in the model. In the CRP mining proposal, the mining block covers an area of 5 km × 2 km. For the purposes of this study, we conceptualize the near‐field area to extend approximately 0.5 km from the mined area and the far‐field to extend to 5 km from the mined area. However, it is important to note that the size of the near‐field and far‐field is relative to the scale of disturbance (usually defined by the extent of detectable impacts) and will therefore vary for other types of disturbances and areas. As our model is not spatially explicit, we assume homogeneous impacts inside all spatial domains.

We estimated the impact on benthic fauna as a decrease in abundance relative to the predisturbed state. For most faunal groups, this decrease in abundance translates to mortality, but as our model also includes mobile fauna that may leave the area but are not killed by the disturbance, we use the term “decrease in abundance” throughout the paper. As a simplification, we only assess decrease in abundance, although some faunal groups may temporarily increase in abundance after seabed disturbance (e.g., Bigham et al., 2023; Pranovi et al., 2000). We divided the abundance variables into two time steps: immediately after disturbance and one year after disturbance based on the available data from the ROBES experiments (Figure 2). We separated the decrease in abundance immediately after mining into direct and indirect decrease in abundance (see Appendix S1 for full description of the modeling framework). Within this framework, some of the organisms will be removed during the direct extraction process (direct impact), depending on the mining efficiency and depth. The remaining fauna will be exposed to indirect impacts (in our model, these are sedimentation and impacts from toxic substance release) that will describe the acute impacts on them (details in Appendix S1, following Kaikkonen, Helle, et al., 2021). The division of direct and indirect pressures that affect the decrease in abundance allows us to evaluate the impacts of mining in both the mined and unmined areas within the same model. As a simplification, we consider that any changes in the seafloor environment (e.g., sediment composition, nodule removal) will only affect the organisms one year after the disturbance, not immediate changes in abundance. The impacts after one year were estimated as the relative change in abundance from the predisturbed state and depend on the initial impacts immediately after disturbance, recovery potential of the functional group, and changes in the habitat quality (such as changes in sediment characteristics and food availability). The affinity to substrate of the different functional groups was considered when making the impact estimates (e.g., sessile invertebrate megafauna are more sensitive to loss of the hard substrate (including nodules) than infauna, see Appendix S1 for details).

### Application: Disturbance scenarios and use of the BN model

BNs enable evaluation of various scenarios and computation of posterior probabilities based on new knowledge (Pearl, 1986). Through BNs, operational parameters can be modified to analyze the effects of different types of seabed mining (or other types of seabed disturbance) and their impact on benthos. The joint probability distribution in the BN can be used to query the effects of multiple pressures on specific ecosystem components, assess associated risks, and identify the variables that should be monitored for an improved understanding of the impacts (Carriger et al., 2016).

In order to assess how changes in the magnitude of disturbance affect benthic fauna, we queried the network on two alternative mining scenarios. These scenarios, which we define as a combination of specific states of the decision variables that describe the overall mining process, are assumed to be controlled by the mining operator (Table 3). In the first scenario, hereafter “High disturbance,” the entire mined area (Figure 2) was disturbed, and sediments were disrupted to deeper than 30 cm. For the second scenario, hereafter “Intermediate disturbance,” 50% of the mined area was disturbed, and sediments were disrupted to a depth of less than 10 cm. In this scenario, a larger area with nodules will remain unmined and will only be affected by sedimentation. The high disturbance scenario was defined by experts based on the description of a proposed mining operation for phosphorite nodules on the Chatham Rise (CRP, 2014), while the intermediate disturbance scenario was based on the anticipated disturbance from other types of mining operations (e.g., surface nodule extraction as proposed for polymetallic nodule mining, e.g., Muñoz‐Royo et al., 2022) and could also be applied to describe low‐penetration bottom trawling (Eigaard et al., 2016). All the other variables in the model are further affected by these decision variables. Note that the model can be queried for any combination of variables; we have presented only a limited number of possible outcomes.

**TABLE 3:** States of the operational variables associated with the two seabed mining disturbance scenarios.

Scenario	Operational variables			
	Area disturbed inside the mining block	Depth of sediment disturbance	Plume release technique	Description
High disturbance	100%	>30 cm	At the seafloor	High impact seabed mining operations
Intermediate disturbance	50%	<10 cm	At the seafloor	Surface collector operations

**FIGURE 3:**
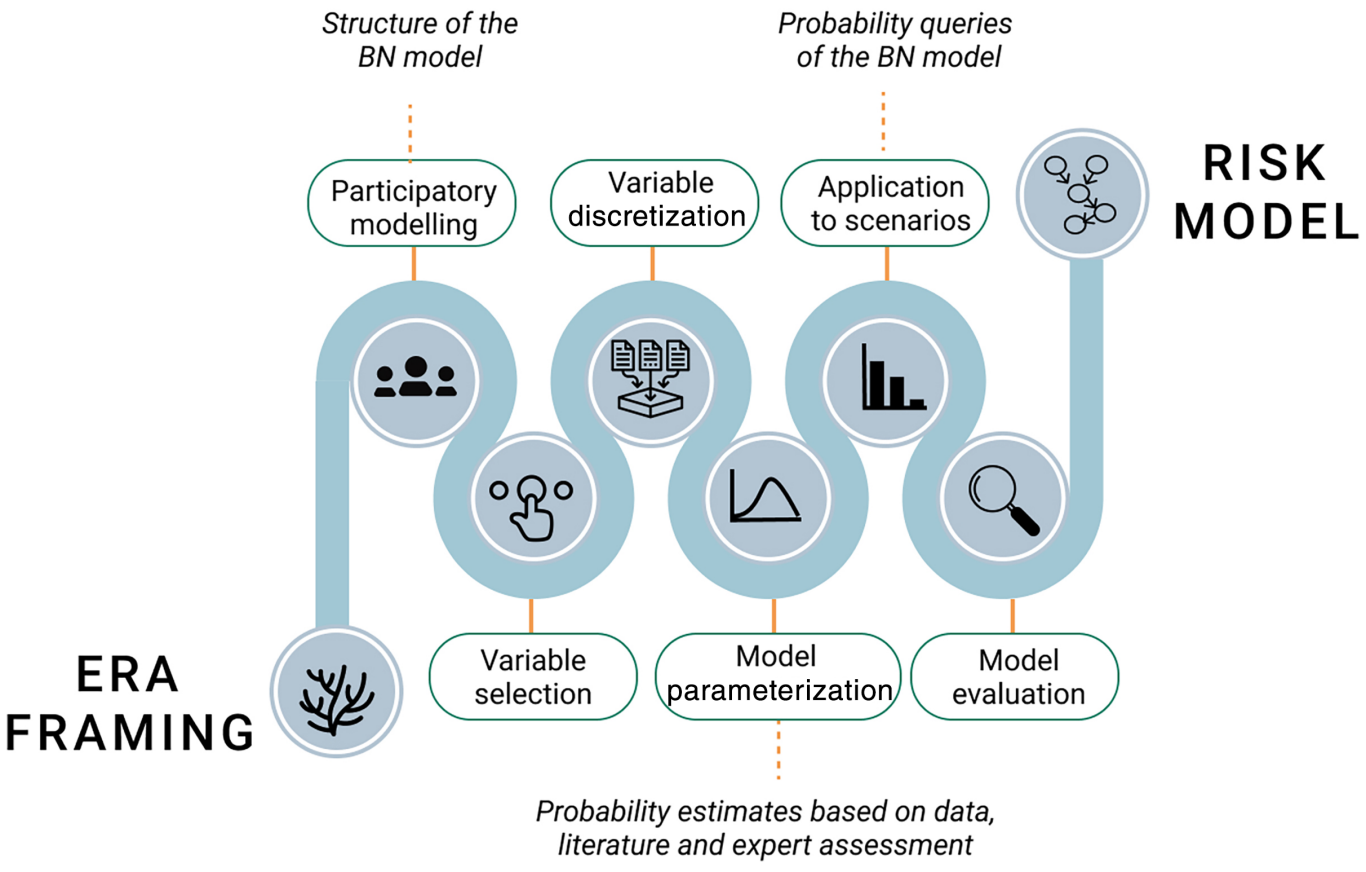
Overview of modeling process. BN, Bayesian network; ERA, environmental risk assessment. Image by Laura Kaikkonen, with all icons either created by Laura Kaikkonen or openly available from the Noun Project under a CC BY license that allows reuse.

Developing models in data‐limited settings presents a challenge for validating these models using conventional statistical methods. This difficulty arises from the impossibility of testing the model against an independent dataset that was not employed during the model's development and quantification process. In addition, conventional sensitivity analyses do not provide much insight as the model structure has been defined by experts. Therefore, the BN was qualitatively evaluated in a series of meetings attended by experts, during which the model and its outcomes were presented, discussed, and agreed upon. A full overview of the model building and application is given in Figure 3.

## RESULTS

**FIGURE 4:**
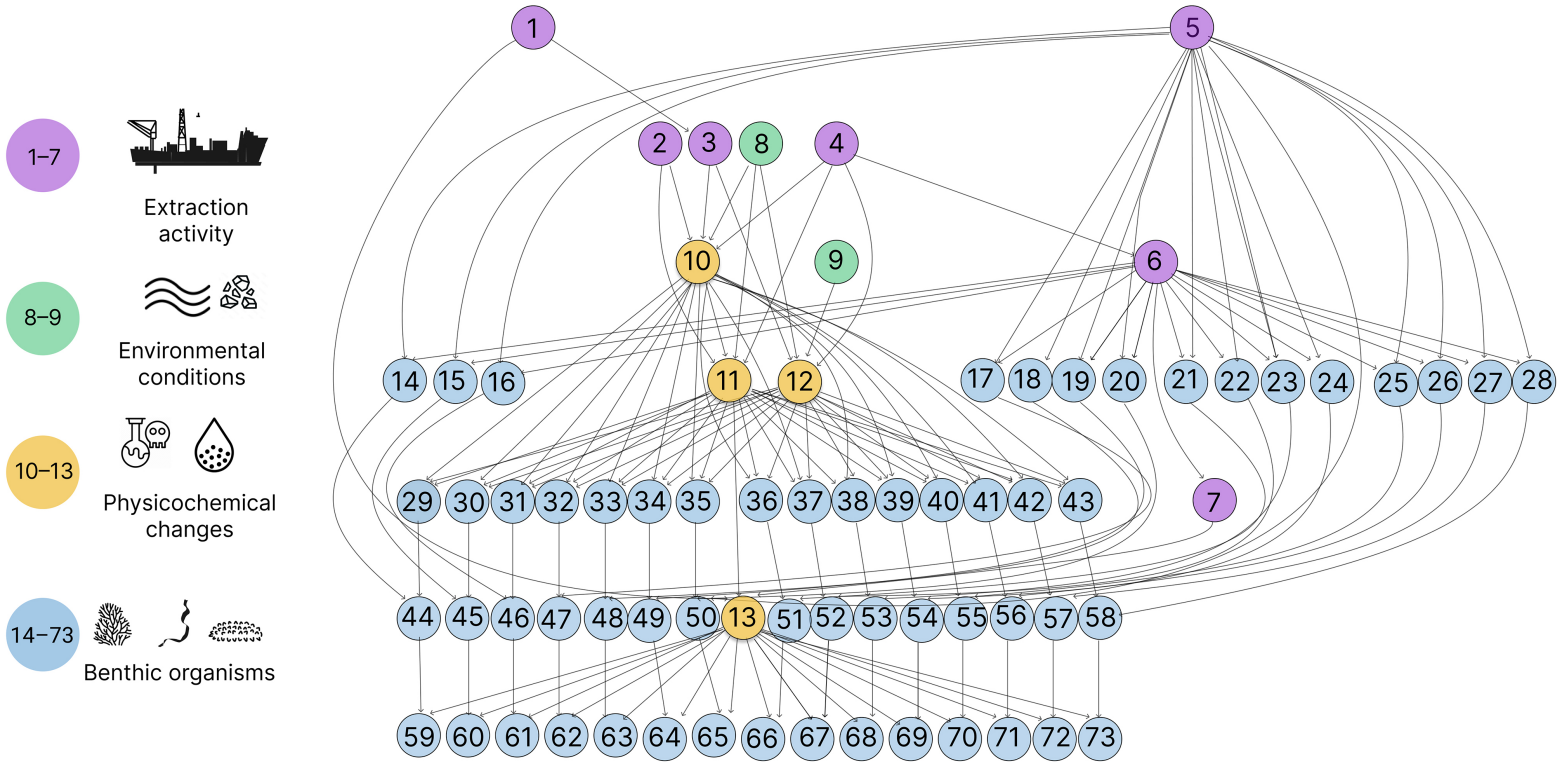
Bayesian network model for risks of seabed mining on benthic fauna, showing the separate variables for the time steps and all functional groups. Purple circles denote operational variables from the two mining scenarios, yellow circles are pressures arising from mining, green circles are environmental conditions (independent of the mining operation), and light blue circles depict the abundance of the benthic fauna in the different functional groups across the four time steps in the Bayesian network model. The numbers shown are for illustration purposes; full details of the model structure and connection between variables can be found within the data and code (https://github.com/lkaikkonen/CR‐ERA). Image by Laura Kaikkonen, with all icons either created by Laura Kaikkonen or openly available from Iconscout and the Noun Project under a license that allows reuse.

The causal mapping and model building process resulted in a BN model for the Chatham Rise with 73 variables and 154 connections (Figure 4). The model has seven independent variables describing the two disturbance scenarios and environmental conditions/pressures caused by mining that further cascade down to responses in benthic fauna. In this section, we present results on the joint probabilities queried on the two disturbance scenarios for the Chatham Rise environment.

### Likelihood of pressures from mining

**FIGURE 5:**
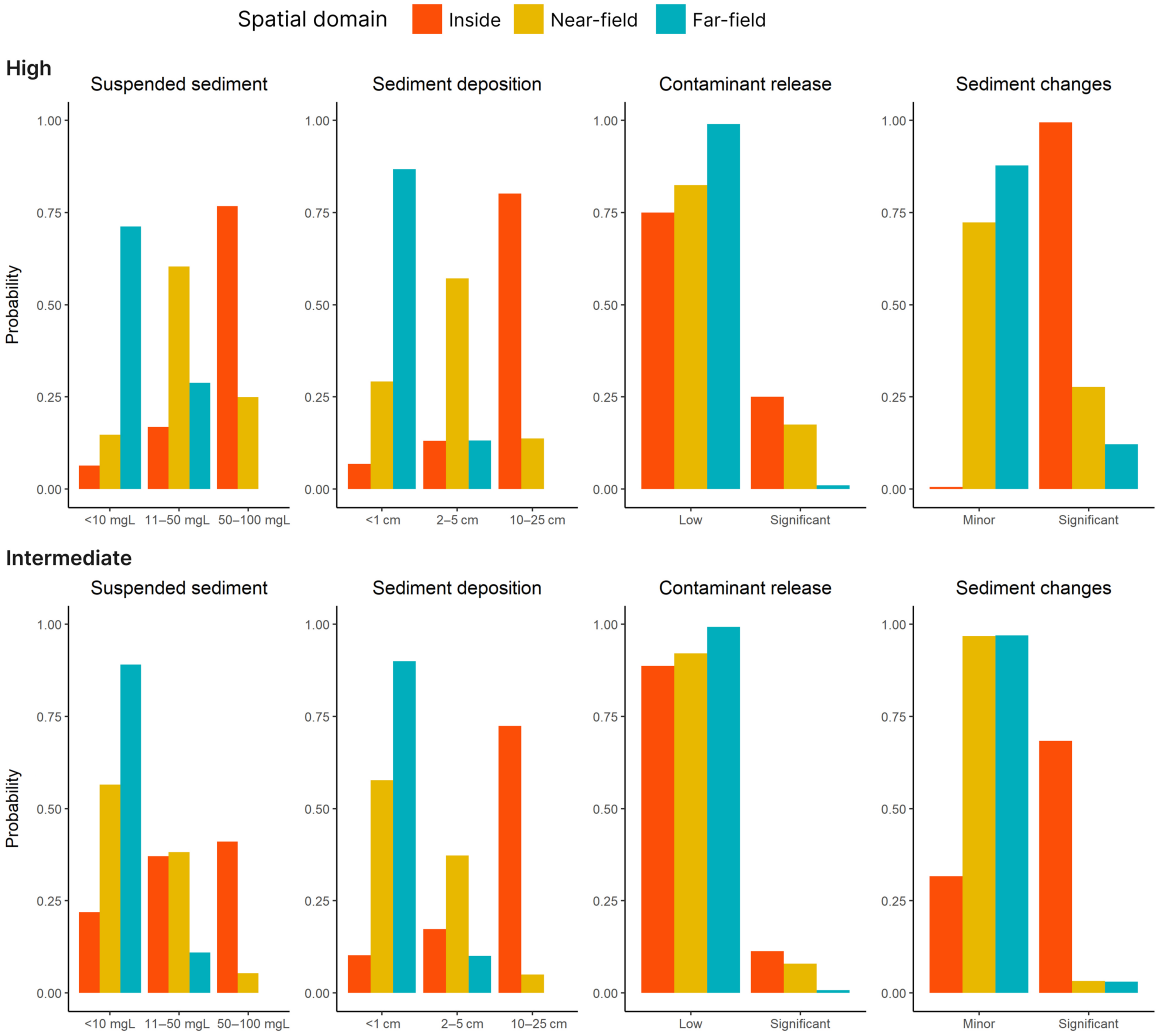
Probability of the different levels of suspended sediment concentration, sediment deposition, contaminant release, and sediment changes resulting from mining inside the mined area, and the near‐field and the far‐field outside of the mining block under the two disturbance scenarios (high and intermediate).

The BN model successfully captured the variation in the likelihood of disturbance from the two different disturbance scenarios assessed (Figure 5). As the highest pressures were confined to inside the mined area, largest differences between the scenarios were predicted inside the mined area and in the near‐field adjacent to the mining block. The probability of different levels of sediment deposition varied as a function of the distance from the mined area and between the two disturbance scenarios. The most likely outcome under both scenarios was high deposition inside the mining block and low deposition in the far‐field. For suspended sediment, moderate SSCs were the most likely outcome inside the mined area and in the near‐field area. In the far‐field, low SSC levels were the most likely. The magnitudes of SSCs and sediment deposition were estimated to be lower under the intermediate disturbance scenario.

The largest difference between the two evaluated scenarios was the probability of significant sediment changes. Under the high disturbance scenario, significant sediment changes were expected not only inside the mined area, but also in the near‐field and in the far‐field. Under the intermediate disturbance scenario, significant sediment changes were confined inside the mining area. As the release of harmful substances was set to be mostly driven by potential concentration of toxic substances in sediment and the sediment substrate type in the model, there was only a small difference between the two scenarios for toxin release. The probability of significant contaminant release from the sediment was low under both scenarios (Figure 5).

### Impacts on benthic fauna

The various functional groups were assigned differential responses to the direct impacts, and subsequent recovery from mining, based on data and expert assessments (Table 4). Mobile epifauna and hyperbenthic species are to an extent able to escape the physical disturbance and thus experience lower decreases in abundance from the direct impacts of mining, whereas sessile fauna inside the mining area will be removed by the sediment extraction. Aside from hyperbenthos, meiofauna were estimated to be most tolerant to indirect impacts of mining. Small macroinfaunal species were estimated to experience moderate to high decreases in abundance from indirect impacts even under intermediate disturbance but had moderate recovery potential after one year. Sessile epifauna, such as stony corals, were estimated to experience high decreases in abundance from direct disturbance, and moderate decreases in abundance from indirect disturbance, but recovery will be limited. However, small soft‐bodied sessile taxa may have the potential to recolonize the area within one year. In the following section, we present a selection of results of the impacts on benthic fauna, conditional on the mining scenarios and the probability of the magnitude of the ecosystem pressures, as presented above. Full results on the impacts on all functional groups are presented in Appendix S2.

**TABLE 4:** Overview of the impacts of seabed mining on benthic fauna underpinning the conditional probability estimates.

Functional group	Direct impact	Indirect impact	Impacts after 1 year
Meiofauna (surface and deep meiofauna)	All organisms in the mining device's path removed	Relatively low decrease in abundance even under high disturbance, sediment deposition has largest impact	Moderate recovery potential
Mobile macroinfauna (small and large)	All organisms in the mining device's path removed	Moderate‐high decrease in abundance under high disturbance, sediment deposition has largest impact	Low/moderate recovery potential
Sessile macroinfauna (small and large)	All organisms in the mining device's path removed	Moderate‐high decrease in abundance under high disturbance, sediment deposition has large impact but SSC most important for this group	Low recovery potential
Hyperbenthos (grazing/deposit feeding, scavenging/predatory)	Most organisms in the mining device's path removed, small portion can escape	Suspended sediment has largest impact	High recovery potential (via recolonization)
Mobile epifauna (grazing/deposit feeding, scavenging/predatory)	Most organisms in the mining device's path removed, small portion can escape	Sediment deposition has largest impact	Some recovery via recolonization, grazing, and scavenging epifauna may benefit from the increased food availability on the disturbed sediment
Sessile epifauna (erect suspension feeders, encrusting suspension feeders, erect filter feeders, encrusting filter feeders, small soft‐bodied megafauna)	All organisms in the mining device's path removed	High decrease in abundance even under moderate disturbance. Sediment deposition and SSC both have a large impact; encrusting forms more prone to sediment deposition than erect ones, filter feeders more tolerant to SSC than suspension feeders	Soft‐bodied small organisms recover quickly even within one year. In other groups no recovery or only marginal recovery potential if no significant sediment changes. In case of complete nodule removal in the mining area, recovery is not possible.

*Note*: The variables (Table 3) included in each functional group are given in brackets.

Abbreviation: SSC, suspended sediment concentration.

#### Immediate impacts under the high and intermediate disturbance scenarios

**FIGURE 6:**
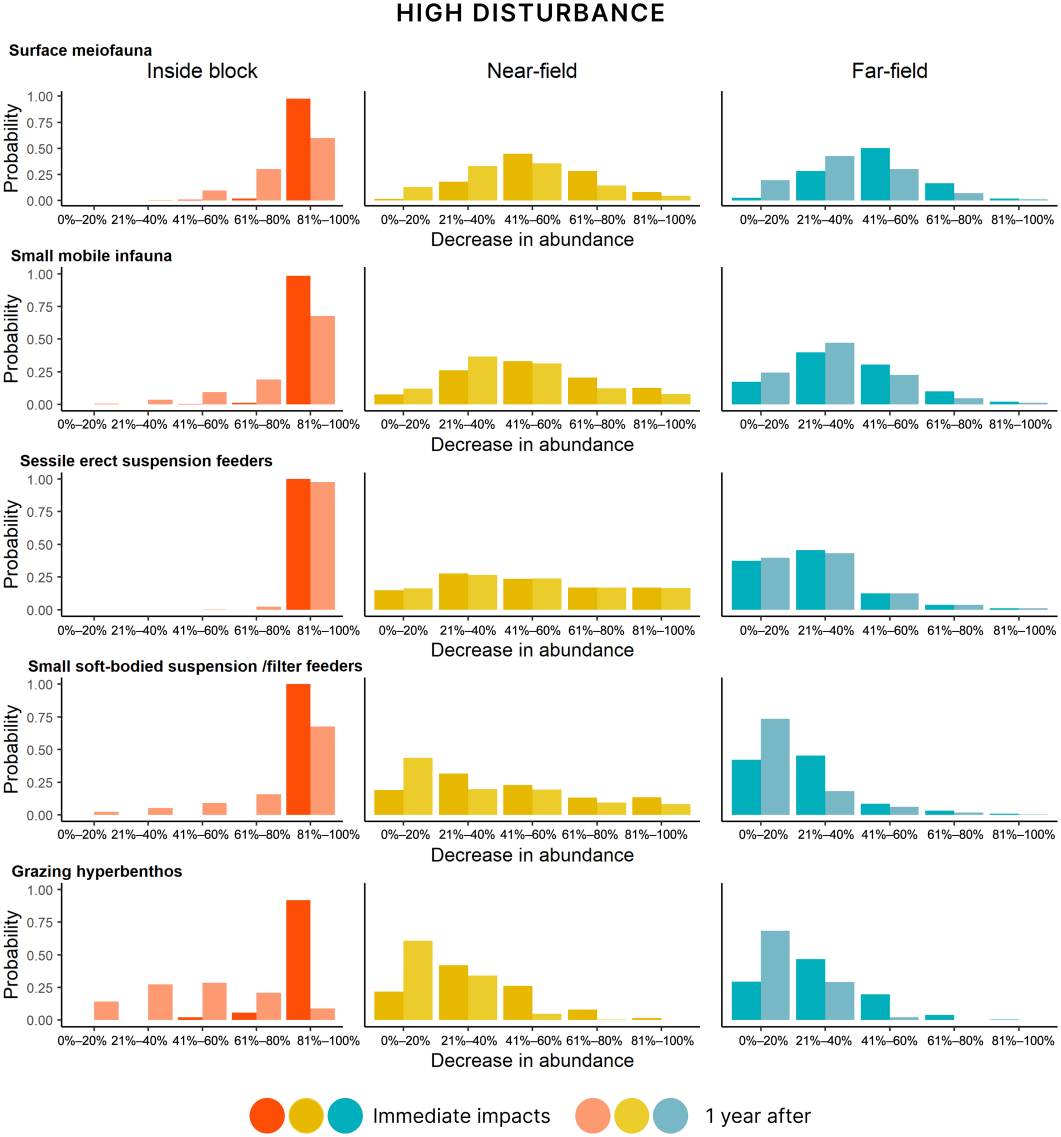
Probability of relative decrease in abundance of five selected epifaunal functional groups inside the mining block (left panel), in the near‐field area directly adjacent to the mined area (middle), and in the far‐field area outside the mining block (right panel) under the high disturbance scenario. Immediate impacts are noted in a dark shade and impacts after one year in a lighter shade. Full results for all functional groups are presented in Appendix S2.

**FIGURE 7:**
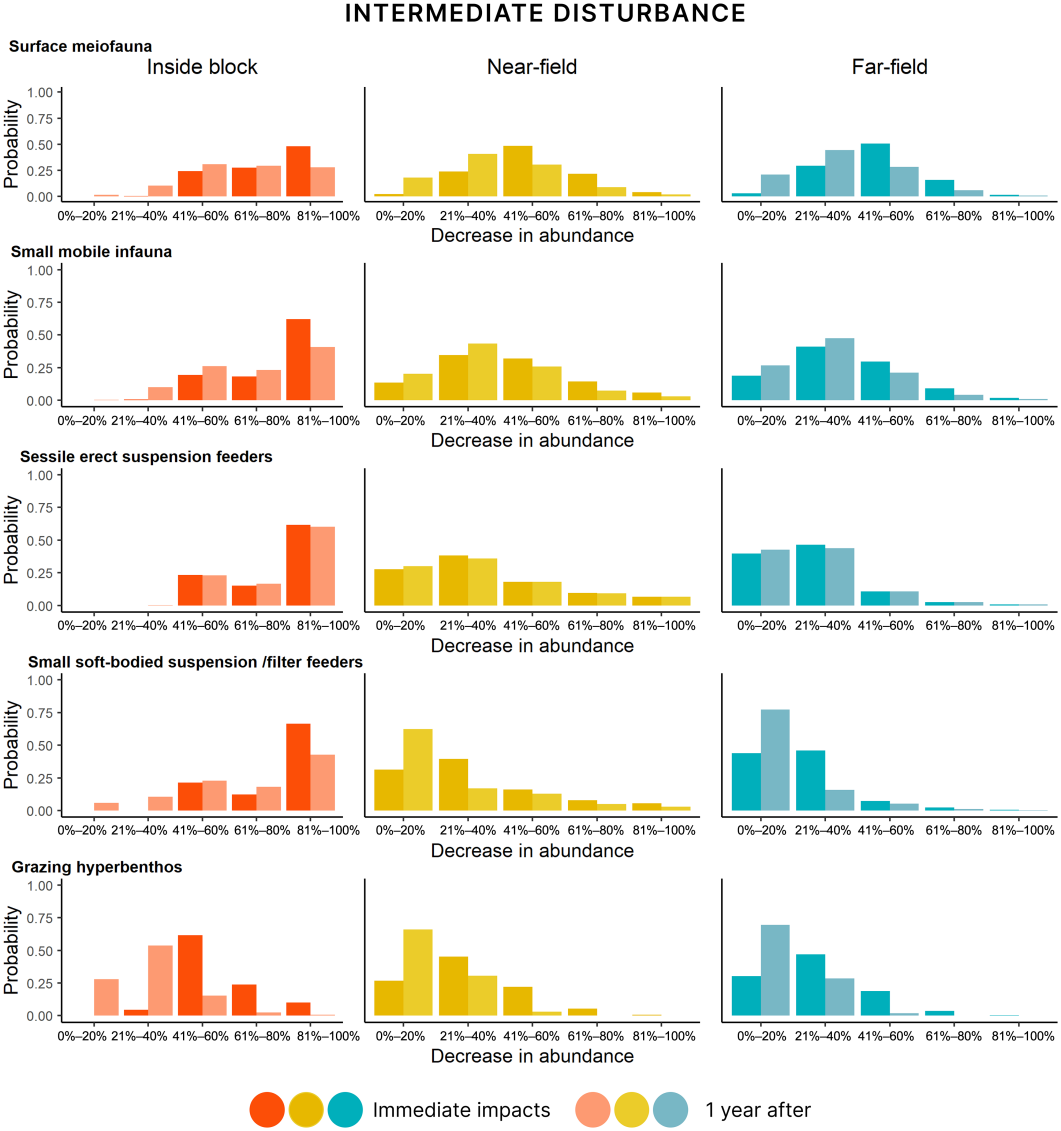
Probability of relative decrease in abundance of five selected epifaunal functional groups inside the mining block (left panel), in the near‐field area directly adjacent to the mined area (middle), and the far‐field area outside the mining block (right panel) under the intermediate disturbance scenario. Immediate impacts are noted in a dark shade and impacts after one year in a lighter shade. Full results of all functional groups are presented in Appendix S2.

The decrease in abundance immediately after the mining disturbance varied between the functional groups and with proximity to the mined area (Figures 6 and 7, full results for all groups in Appendix S2: Figures S1–S6). Despite the moderate to low levels of physicochemical disturbance (Figure 5), most benthic organisms, regardless of their functional group, were predicted to decrease in abundance by 60%–100% inside the mined area and by 20%–60% in the unmined near‐field. The highest levels of relative changes in abundance in the far‐field were for encrusting sessile suspension and filter feeders. Under the intermediate disturbance scenario, changes in abundance were lower and there was more variation in the potential responses of fauna inside the mined area (Figure 7). In the near‐field, under this scenario, 20%–40% decrease in abundance was the most likely outcome for most groups. In the far‐field area, the differences between the two scenarios were smaller. Importantly, we noted increasing levels of uncertainty in the estimates for biological responses with increasing distance from the mined area and relatively lower levels of pressures from mining.

Highest changes in abundance were estimated inside the mined area under both scenarios, where all sessile and infaunal organisms were estimated to experience high relative decrease in abundance (81%–100% compared with the predisturbance community) (Figures 6 and 7). In the near‐field, the most likely outcome under the high disturbance scenario was 41%–60% reduction in abundance for all macroinfaunal groups. The changes in abundance in the near‐field were lower under the intermediate disturbance scenario (21%–40% decrease in abundance). In the far‐field, for most macroinfaunal groups, the most likely outcome was 21%–40% decrease in abundance. Mobile epibenthos and hyperbenthic organisms had the lowest decrease in abundance in both the far‐ and near‐field areas under both disturbance scenarios (Figures 6 and 7), with the smallest decrease in abundance predicted for mobile organisms in the far‐field area.

#### Impacts after one year

In our model, the probability of decrease in abundance one year following seabed disturbance is conditional on the initial disturbance‐related decrease in abundance and changes in the environment. The estimates of relative decrease in abundance one year after disturbance therefore incorporate both a metric of recovery and any additional decrease due to sublethal effects (see Table 4; Appendix S1 for details). Recovery was estimated to be more likely across all faunal groups under the intermediate disturbance scenario, which resulted in overall lower changes in abundance (Figure 7) and a lower likelihood of significant sediment changes (Figure 5). Recovery was more likely to occur in the near‐field and the far‐field, compared with inside the mined area, as there was less likelihood of significant sediment changes. Similarly, recovery was more likely under the intermediate disturbance scenario when a larger proportion of the original abundance remained and the sediment changes were smaller, compared with the high disturbance example (Figure 6).

Sessile epifauna were the least likely to show recovery after one year (Figures 6 and 7). The most likely outcome inside the mined area for most sessile megafauna was 80%–100% decrease in abundance, with likelihood of high decrease in abundance under the high disturbance scenario (Figure 6) than the intermediate disturbance scenario (Figure 7). An exception within this group were the soft‐bodied megafauna, which were deemed to have recovered nearly fully (0%–20% decrease in abundance compared with predisturbed state) in the near‐ and far‐field under both scenarios. In turn, mobile benthos were estimated to be the least affected: in the near‐field and far‐field, small to no or little decrease in abundances (21%–40% and 0%–20% change, respectively) of hyperbenthos were expected after one year.

#### Comparison of scenarios

**FIGURE 8:**
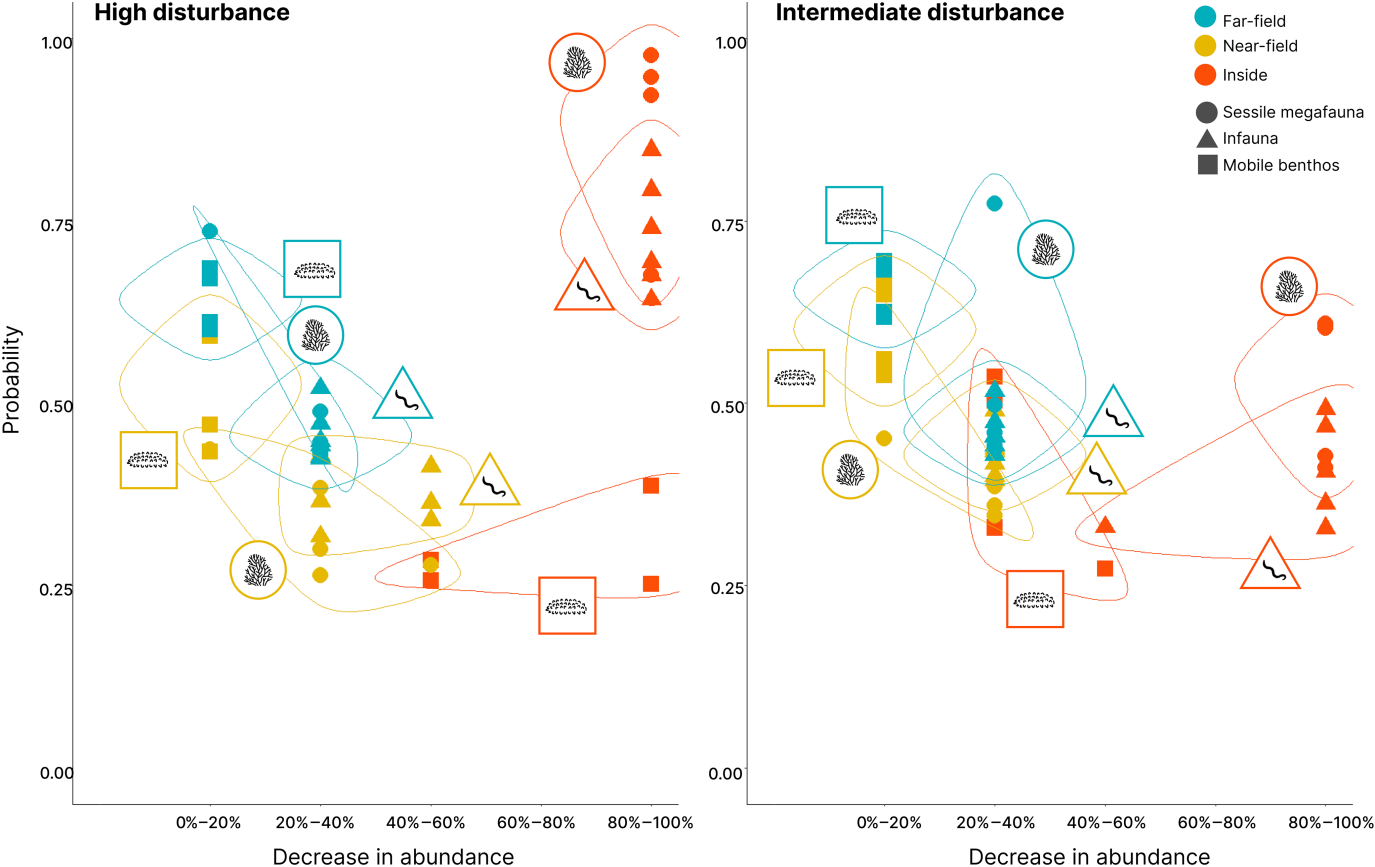
Summary of the most likely outcome for the functional groups in each spatial domain after one year under the high (left panel) and intermediate disturbance (right panel) scenarios. The points in the scatterplot represent the most probable outcome for each functional group as a function of its associated probability. The colors depict the three spatial domains (inside minds area, near‐field, far‐field), and the broad faunal groups (Functional group: Sessile megafauna, infauna (meio and macro) and mobile invertebrate megafauna) are shown in different shapes and icons. Image by Laura Kaikkonen, with all icons either created by Laura Kaikkonen or openly available from Iconscout and the Noun Project under a license that allows reuse.

Inside the mined area, differences between the two disturbance scenarios after one year were particularly evident for mobile benthos, for which the most likely outcome was a 40%–100% decrease in abundance under the high disturbance scenario, as opposed to 20%–60% under the intermediate disturbance scenario (Figure 8). In the far‐field, the differences between the two scenarios were very small for most functional groups, particularly the sessile megafauna. As the probabilities of high impact in the far‐field were lower under the intermediate disturbance scenario, there is less difference between these two spatial domains compared with the differences observed under the high disturbance scenario.

Under the high disturbance scenario, the probability estimates for the near‐ and far‐field were more variable. Overall, for both scenarios, highest certainties were given to impacts inside the mined area.

### Model validation and sensitivity

The expert group was satisfied with the BN model's ability to capture the variation in the impacts across the different seabed mining disturbance scenarios and spatial domains. Reviewing the model results showed that mining intensity had the highest impact on the magnitude of physicochemical pressures. As both the model structure and the parameters are largely expert informed, there was no need to perform a numerical sensitivity analysis to evaluate which variables had the highest impact on the final outcome (as reflected in a decrease in abundance of benthic fauna).

The use of probability distributions in BNs allow the uncertainty regarding the variation in a variable of interest to be directly embedded in the impact estimates. To account for the uncertainty resulting from the lack of information on the process being evaluated, we also recorded the experts' certainty in the probability estimates (Table 5).

**TABLE 5:** Overview of the confidence in the variable parameterization.

Variable group	In situ data	Experimental data	Literature	Confidence
Sedimentation (SSC and sediment deposition)	Yes	No	Yes (Lescinski et al., 2014)	Moderate
Contaminant release	No	No	Yes (Frontin‐Rollet, 2017; Hauton et al., 2017; Simon et al., 2022)	Low
Mobile megabenthos and hyperbenthos	No	No	None (baseline information in Lörz, 2011)	Low
Meiofauna	Yes	No	Yes (in situ data published in Leduc et al., 2024)	Moderate
Macroinfauna	Yes	No	None	Moderate
Sessile megafauna	No	Yes	Yes (Bell et al., 2015; Brooke et al., 2009; Leys, 2013; Mobilia et al., 2021, 2023; Pineda et al., 2017; Wurz et al., 2021)	Moderate

*Note*: Full details of information used are included in Appendix S1.

Abbreviation: SSC, suspended sediment concentration.

For the physicochemical parameters, highest uncertainties were assigned to potential release of harmful substances from the sediment and extracted phosphatic mineral material (Table 5). Similarly, the impacts of toxin release on all groups of benthic fauna were ranked as highly uncertain, as few studies have been published on the topic and the number of potentially harmful substances is unknown. For this reason, the importance of toxin release received a low weight in the impact estimates for all benthic functional groups, but this should be considered in future ERAs as a potentially significant stressor.

The highest uncertainties within functional groups were assigned to mobile epibenthic organisms and hyperbenthos (Table 5). This uncertainty is also reflected in the probability estimates, where hyperbenthos estimates are the most varied (broadest distribution). In turn, meiofauna and small mobile macroinfauna received highest certainty, as for these taxa both new data and previous studies could be used to assess the impacts (Tables 2 and 5).

## DISCUSSION

Uncertainty regarding biological responses of marine organisms to seabed disturbance is a major concern for estimating the impacts of human activities in the deep sea, which subsequently impacts decision‐making and policymaking (Kung et al., 2021). To quantify the uncertainties in biological responses, we developed a probabilistic ecological risk assessment model to describe the pressures caused by deep seabed mining and the responses of affected benthic ecosystem components. We estimated the magnitude of the ecosystem responses and probability of recovery by combining field and experimental data, information from published literature, and expert knowledge. This type of model can be used to identify the likelihood of ecosystem losses from seabed disturbance to guide the regulation and management of such activities.

Most benthic functional groups evaluated in this study were anticipated to tolerate the low levels of sedimentation relatively well and be better adapted to moderate temporary increases in suspended sediment than those in more stable systems (such as abyssal plains). However, the results of our modeling indicated that most benthic functional groups of the Chatham Rise were expected to decrease in abundance and would not recover even from intermediate disturbance outside the mined area after one year. There is high uncertainty around predicted impacts, especially outside mined seafloor areas, and the results stress the importance of further studies on the recovery dynamics at broader spatial and temporal scales.

There are important considerations when applying the approach and the results presented here to other deep‐sea ecosystems and forms of disturbance (such as regions where abyssal manganese nodules, or placer or dredged deposits may be extracted from the seafloor). While the results give some insights to the broad patterns of how deep‐sea benthic organisms may respond to seabed disturbance, the magnitude of pressures and the responses of biological communities are likely to vary considerably from one area to another (e.g., Boschen et al., 2013; Haffert et al., 2020; Jones et al., 2017). Applying similar quantitative ERA models in other areas where deep‐sea mining is considered, such as the Clarion‐Clipperton Zone, is therefore important, as it enables a systematic and data‐driven evaluation of potential risks, environmental impacts, and uncertainties across multiple habitats associated with this emerging industry.

### Incorporating ecological data into ERAs

Despite an increase in research regarding the impacts of seabed disturbance to seafloor ecosystems (e.g., Gollner et al., 2017; Jones et al., 2017), data are rarely comprehensive enough and cover all relevant ecosystem components to enable evidence‐based decision‐making and robust environmental management of human activities (Amon et al., 2022). To overcome the inherent data paucity in many deep‐sea environments, it is necessary to use all possible scientific evidence, from other industries (e.g., Kaikkonen et al., 2018) as well as analogies to shallow‐water systems and communities (Van Der Grient & Drazen, 2021). Our approach in combining empirical data and expert assessment demonstrates that, despite the considerable body of literature on the different aspects of physical and sedimentation impacts, formulating conclusions on the impacts is not an easy task. In light of these challenges, the probabilistic approach as employed here proved useful, as the uncertainties related to the impacts were directly incorporated in the impact estimates. We found that experts were more comfortable giving uncertain judgments when this aspect was embedded in the process. Furthermore, the approach provides a method to synthesize information from multiple sources and move from qualitative risk statements to more quantitative impact estimates. As the conditional probabilities may be drawn from multiple sources, the model can be continuously updated as new information becomes available and be tailored specifically to a proposed method of exploitation (see Table 5 for used information sources and evidence gaps).

A major issue with determining the sensitivity of species or groupings of functionally similar organisms to environmental disturbance is that most organisms are poorly studied, thus detailed information on the ecological responses is limited outside some specific habitats such as hydrothermal vents (Chapman et al., 2019). Nevertheless, trait‐based approaches are useful in identifying species responses to direct anthropogenic impacts and other environmental changes (Baird et al., 2008; Boschen‐Rose et al., 2021; Krumhansl et al., 2016; Lundquist et al., 2018). We found that generalizing biological responses using broad functional groups allows for a pragmatic understanding of how organisms may respond to external stressors (Miatta et al., 2021). The use of broad functional groups facilitates the application of data collected from one area to another, although in such cases, the variation in the trait expressions associated with biological responses across regions must be carefully evaluated (de Juan et al., 2022).

### Improving probabilistic ecological risk assessments

Human activities may result in multiple changes in the environment with different spatial and temporal scales, and as a result, modeling such complex systems with any method comes with drawbacks and often requires simplification to enable a satisfactory result (e.g., Uusitalo, 2007). Handling many variable connections, impact pathways, and associated evidence is a demanding task, and ensuring that all experts participating in the assessment understand the study background is a challenge. The complexity of integrating different types of data and knowledge (biological, technical, geological, oceanographic) into the chosen model not only poses communication challenges for the assessment team and between experts, but handling and organizing the collected information require considerable effort by the experts and the modelers. While the workload involved in quantifying a large BN model may seem overwhelming, a similar (or larger) workload applies to any kind of impact assessment that incorporates and assesses complex connections between ecosystem components.

Aside from the workload involved in parameterizing large BNs, another drawback of using BNs is their acyclic nature, preventing the inclusion of feedback loops between parent and child nodes (e.g., for ecosystem interactions; Uusitalo, 2007). This issue can be partially overcome with the use of splitting nodes, or through Dynamic BN approaches with multiple time steps (Trifonova et al., 2015). As another technical improvement, moving to hybrid BNs that allow mixing continuous and discrete variables would provide opportunities to describe the impacts more precisely without the need to simplify potential impacts to discrete classes (e.g., Moe et al., 2020).

There are several ways the BN model developed here can be augmented to be more useful in an ERA context. First, improving recovery estimates of the geochemical and biological components in the model would be beneficial to account for the magnitude of the disturbance in the recovery estimates. Second, an important addition that would make the approach more directly relevant to a proposed operation would be combining this model with spatially explicit data, such as sediment plume modeling, to estimate the spatial extent of the SSCs and sediment deposition from the mining activities as a function of distance from the mined area (Lescinski et al., 2014; Spearman et al., 2020). A similar approach can be used if detailed spatial information on the biological communities potentially affected by the activity is available (e.g., Helle et al., 2020), enabling a more precise spatial representation of the risks. Third, in a real‐world case, the ERA should consider impacts on a broader set of ecosystem components and their interactions. Even if the pelagic realm remains more poorly studied than seafloor ecosystems (Bisson et al., 2023; Robison, 2004), improving estimates on mobile fauna (Washburn et al., 2023) and including other groups of organisms, such as microorganisms (Herndl & Reinthaler, 2013), plankton (Stenvers et al., 2023), and fishes (Drazen et al., 2020), is essential to fully assess the extent and magnitude of mining or sediment disturbance. Finally, our approach does not include detailed information on the full interactions within the ecosystem. For example, long‐term (multi‐decadal) deleterious impacts on ecosystem functioning have been demonstrated in disturbance experiments in abyssal nodule fields (e.g., Peru Basin, Vonnahme et al., 2020), and in the future, it will be important to examine the interplay between different ecosystem components and biogeochemical linkages.

### Further applications

Environmental management often requires decision‐making under uncertainty regarding the potential outcomes of activities and the most effective ways to mitigate them. Despite recommendations for the use of probabilistic methods in risk assessments (Van den Brink et al., 2016), their comprehensive integration into regulatory risk frameworks is still limited. Deterministic approaches, such as calculating single risk values based on a predicted exposure to a stressor remain more prevalent (Fairbrother et al., 2016). By utilizing a probabilistic model capable of generating estimates for various scenarios, it would be feasible to identify management actions that are most likely to minimize stressor inputs in the context of impacts of human activities or environmental changes leading to improved chances for the maintenance of the ecological functions of impacted deep‐sea faunal communities.

The probabilistic model described in this study was developed, from a scientific perspective, to provide a framework for further applications. For a real‐world application for management purposes, it is important to engage with the relevant regulatory bodies and stakeholders to ensure that the model framing and metrics align with societal and management needs (e.g., specific species and habitats or maximum thresholds for allowed impacts). For management purposes, a useful quality of BN models is that they may be further augmented to incorporate socioeconomic data (Uusitalo et al., 2022) and consider broader complex interactions, for instance, in the context of climate change effects (Furlan et al., 2020). To ensure the optimal use of the models, such risk assessments should involve interdisciplinary collaboration between a diverse group of scientists, policymakers, and stakeholders to ensure that the best available knowledge is integrated into the decision‐making process.

## CONFLICT OF INTEREST STATEMENT

The authors declare no conflicts of interest.

## Supporting information


Appendix S1.

Appendix S2.

## Data Availability

Data and code (github.com/lkaikkonen/CR-ERA) are available in Zenodo at https://doi.org/10.5281/zenodo.10020255.
