# Supplementary material for: Probabilistic ecological risk assessment for deep‐sea mining: A Bayesian network for Chatham Rise, Pacific Ocean
Source: Ecol Appl. 2024 Nov 25;35(1):e3064. doi: 10.1002/eap.3064 (PMC11734116; doi:10.1002/eap.3064)
Supplement: Supplementary file 1 — Appendix S1. [file EAP-35-e3064-s002.pdf]

Probabilistic ecological risk assessment for deep-sea mining: A Bayesian network for Chatham Rise, Pacific Ocean

Ecological Applications

Laura Kaikkonen, Malcolm R. Clark, Daniel Leduc, Scott D. Nodder, Ashley A. Rowden, David A. Bowden, Jennifer Beaumont, Vonda Cummings

Appendix S1

- Appendix S1 Table S1 Overview of the data collection methods used in the study.
- Appendix S1 S1 Detailed method description
- Appendix S1 Table S2 Example of a conditional probability table
- Appendix S1 Table S3. Experts involved in the modelling process & probability elicitation
- Appendix S1 Table S4. Details of variable discretisation and parameterisation

Table S1 Overview of the data collection methods used in the study.

**Parameter measured**	**Model variable(s) used for**	**Sampling device**	**Sampling strategy / Sample size**
Sedimentation characteristics	Sediment deposition, Suspended solids concentration	Benthic lander with the following instruments:<br>• Acoustic doppler current profiler (Nortek Aquadopp 2 MHz)<br>• Turbidity sensors (an AQUAscat and Aquatec AQUAloggers) Temperature-salinity-dissolved oxygen sensor (Seabird MicroCAT) Camera and light Niskin water bottle (5 litre)<br>• Sediment trap (Technicap, 12 sample bottles) to measure particle deposition.	Three benthic landers were deployed for the duration of the disturbance experiment. The landers were deployed on 15 June 2019 and retrieved on 25 June 2019 for analysis of the samples and data they collected.
Sediment particle size composition	Particle size composition of the extracted sediment	Multicorer	N = 34
Sediment particle size composition	Sediment changes	Multicorer	Pre disturbance<br>N= 26<br>Post disturbance<br>N=26<br>1 year post disturbance<br>N=26
Meiofauna	Surface meiofauna, deep meiofauna	Multicorer	Pre disturbance<br>N= 26<br>Post disturbance<br>N=26<br>1 year post disturbance<br>N=26
Macroinfauna	Small sessile macroinfauna, Small mobile macroinfauna, Large sessile macroinfauna, Large mobile macroinfauna	Multicorer	Pre disturbance<br>N= 26<br>Post disturbance<br>N=26<br>1 year post disturbance<br>N=26
Sessile invertebrate megafauna	Sessile erect suspension feeders, Sessile erect filter feeders	Beam trawl. Corals and sponges exposed to elevated sediment concentrations in aquariums (see details in Mobilia et al. 2021 and Mobilia et al. 2023.)	Corals and sponges were exposed to four different target concentrations of suspended sediment (SSCs): 0, 50, 100, 500 mg l^{-1}, with n = 4 replicate experimental aquaria for each treatment (Mobilia et al., 2021, 2023).

S1 METHODS DESCRIPTION

Step 1: Participatory modelling

The first stage of the work focused on defining which ecosystem components should be included in the risk model and mapping out their interdependencies based on a previous network drawn from literature and expert opinion (Kaikkonen et al. 2021a). This consisted of defining the model structure with experts in semi-structured interviews, which were implemented as a causal mapping exercise, asking experts to identify the impact pathways associated with seabed mining. We held both joint workshops and individual interviews, which were complement by data analysis from the field measurements as well as literature reviews. Model building consisted of first identifying variable related to the mining technology to recognise the pressures caused by mineral extraction, then the relevant environmental conditions that may affect the magnitudes of these pressures, and finally the affected non-biological (e.g., changes in seabed topography, sediment composition), and biological ecosystem components. This step resulted in a generic modelling framework for any type of benthic organism.

Step 2: Variable selection: Assigning functional groups

Based on the general causal network developed in step 1, we defined variables describing the benthic ecosystem components that were likely to be affected by the disturbance from mining. To address the challenge of assessing the impacts of seabed disturbance on the numerous species inhabiting the Chatham Rise, we simplified the process by grouping organisms into functional groups. Our selection of functional groups was guided by traits known to influence biological responses to various mining-induced pressures, using previously created groupings for the Chatham Rise as a starting point (Lundquist et al., 2018). Functional groups in the model were selected based on traits that have been shown to affect impacts to sedimentation and recovery potential of organisms: body size, feeding habit, position in sediment, and mobility (Hewitt, Lundquist, and Ellis 2018). The number of trait expressions used to determine the groupings varied between functional groups. If a trait was not seen to affect the response in some broader group of organisms, all the different combinations of trait expressions were not used.

The final causal network was modelled as a Bayesian network (BN). Bayesian statistics provide an alternative to commonly used simple scoring procedures in ecological risk assessment. For an introduction to BNs, see e.g., Kaikkonen et al. (2021b), Aguilera et al. (2011) and Chen and Pollino (2012).

Step 3: Variable discretization

Discrete variables were defined to describe the variation in the magnitude of pressures arising from the mining activity. This included considering which combinations of pressures the

extraction and sediment deposition are likely to result in. The discrete states may be described through quantitative metrics, like different concentrations of substances or depth in centimetres, or can be based on qualitative descriptions of discrete classes (e.g., high, medium, low). The key aspect with regard to the biological responses was that these pressure levels should make sense from an ecological perspective. As the demonstration in our case study is not bound to a specific setting, we decided to frame the model variables quite generally with the states low-moderate-high. The discrete variable states were mostly defined based on expert judgement, informed by data and literature (see Table S4 for details).

Step 4: Model parameterisation

Within a BN, the magnitudes of impacts are illustrated through conditional dependencies. These describe the strength of the causal relationships between variables in the model. The probabilities of each value of the child node, conditioned on every possible combination of values of the parent nodes are summarised in conditional probability tables (CPT, see example in Table S2). All CPTs used in this study were developed manually by experts using field and experimental data and literature as supporting information (see Table S4). Where data on the impacts were available, we used experimental and field data to inform the probability distributions and complemented these with expert assessment. As BNs require probability estimates for all the combinations of variable states, the configurations of parent variables that were not applied in the field study were estimated by experts through the following procedure.

We used the graphical interface provided in the Application for Conditional probability Elicitation (ACE) application (Hassall et al., 2019) to initialise the CPTs for the geological variables. The application provides a starting point for defining the overall shape of a conditional probability distribution by ranking the direction and magnitude of the parent nodes on the child node (i.e., the expert will define whether a parent node has a positive, negative, or neutral effect on the child node and the relative strength of that influence) and populating the table through a scoring algorithm. This weight-based distribution can them be easily modified by or together with the experts.

For variables describing the impacts on benthic fauna, direct and indirect mortality were modelled separately. This division accounts for the total mortality of benthic fauna, which encompasses both direct mortality resulting from sediment and mineral concretions extraction, and indirect mortality arising from other pressures such as sediment deposition, suspended sediment, and the release of harmful toxic substances. This approach enables us to estimate the effects of these pressures in both the direct mining area (total mortality) and neighbouring areas (indirect mortality). Organisms within the mining block will experience both direct mortality and, in cases where <100% of the block is mined, the remaining fauna inside the block will be affected by sedimentation. Organisms in the near-field and the far-field will only be impacted by sedimentation effects. This separation was achieved by introducing three auxiliary variables to describe changes in faunal abundance:

- Direct impacts
- Indirect impacts
- Total decrease in abundance

The model was set so that direct impacts applied only inside the mining block, and indirect impacts in all domains. We calculated the probability of changes in abundance from the indirect impacts given the initial direct removal inside the mining block using equation (1). Outside the mining block (in the near-field and far-field) all changes in abundance are due to the indirect impacts.

Direct mortality was estimated as a direct proportion of the mined area, so that e.g., mining 50% of an area results in 50% of fauna being extracted.

For the indirect pressures, we elicited the impacts on each faunal group using a combination of data when available and expert knowledge (Table S4). In order to reduce the elicitation burden on experts, we applied an interpolation method (Barons et al. 2022) to derive the probability distributions. The method assumes that the child node can be estimated through a beta distribution and requires the user to identify both a "best case" and a "worst case" distribution for the child node. In this context, "best" and "worst" pertain to distributions where the parents affecting the node are at their highest and lowest points for the child, respectively. The probability distributions for all other combinations of parent variables are then inferred by interpolating between these extremes. This procedure is accomplished using a series of weights assigned to the parent states, which are employed to interpolate between the parameters of a beta distribution.

We elicited the probabilities for the best- and worst-case scenarios using the following questions:

- What is the lowest the value could be?
- What is the highest the value could be?
- What is the most likely value?

For the biological variables describing the impacts on functional groups for which no data were available, we referred to existing data sets from the Chatham Rise to estimate the approximate community composition of the benthic community within the selected functional groups (e.g., the proportion of demosponges-glass sponges) to assist in the probability estimates. Despite the use of the CPT interpolation tool, all CPTs were carefully reviewed to ensure that the experts' views on the potential impacts were correctly captured by the weights given to the parent nodes.

The separate CPTs for direct and indirect impacts were combined so that the total mortality of benthic fauna within a discrete block and one moment in time comprises the direct mortality from extraction of sediment and mineral concretions, and the indirect mortality of the remaining fauna that are exposed to the pressures from the extraction activity (i.e. one organism cannot be

both extracted and die of sediment deposition). The probability of total mortality of benthic fauna was thus calculated as done in (Kaikkonen et al., 2021a):

$$(1)\ P(Total\ mortality) = P(Direct\ Mortality) + P(Indirect\ Mortality)\ x\ (1 - P(Direct\ Mortality))$$

where p(Indirect Mortality) x (1-p(Direct Mortality)) accounts for the probability of the proportion of fauna remaining after direct extraction. While the resulting joint probabilities are continuous, here we calculate them at 1% accuracy (e.g., round them up) to provide the probabilities of a mortality of a given proportion of benthic fauna for the five discrete classes we use in our model.

Table S2. Example of a part of a finalised conditional probability table (CPT) for indirect effects on benthic fauna. The CPT summarises all possible combinations of the different variable states for a given node (full table has 18 rows for all the combinations of parent variable states).

**Suspended sediment**	**Sediment deposition**	**Contaminant release**	**Decrease in abundance**				
			**0-20%**	**21-40%**	**41-60%**	**61-80%**	**81-100%**
Low	Low	Insignificant	0.8928	0.0735	0.0235	0.0095	0.0007
Low	Low	Significant	0.8019	0.1297	0.0548	0.0126	0.001
Low	Medium	Insignificant	0.8306	0.1058	0.0411	0.0156	0.0069
Low	Medium	Significant	0.7001	0.1774	0.0784	0.0354	0.0087
Low	High	Insignificant	0.0146	0.1999	0.3378	0.2561	0.1916
Low	High	Significant	0.0115	0.0861	0.2207	0.3557	0.326

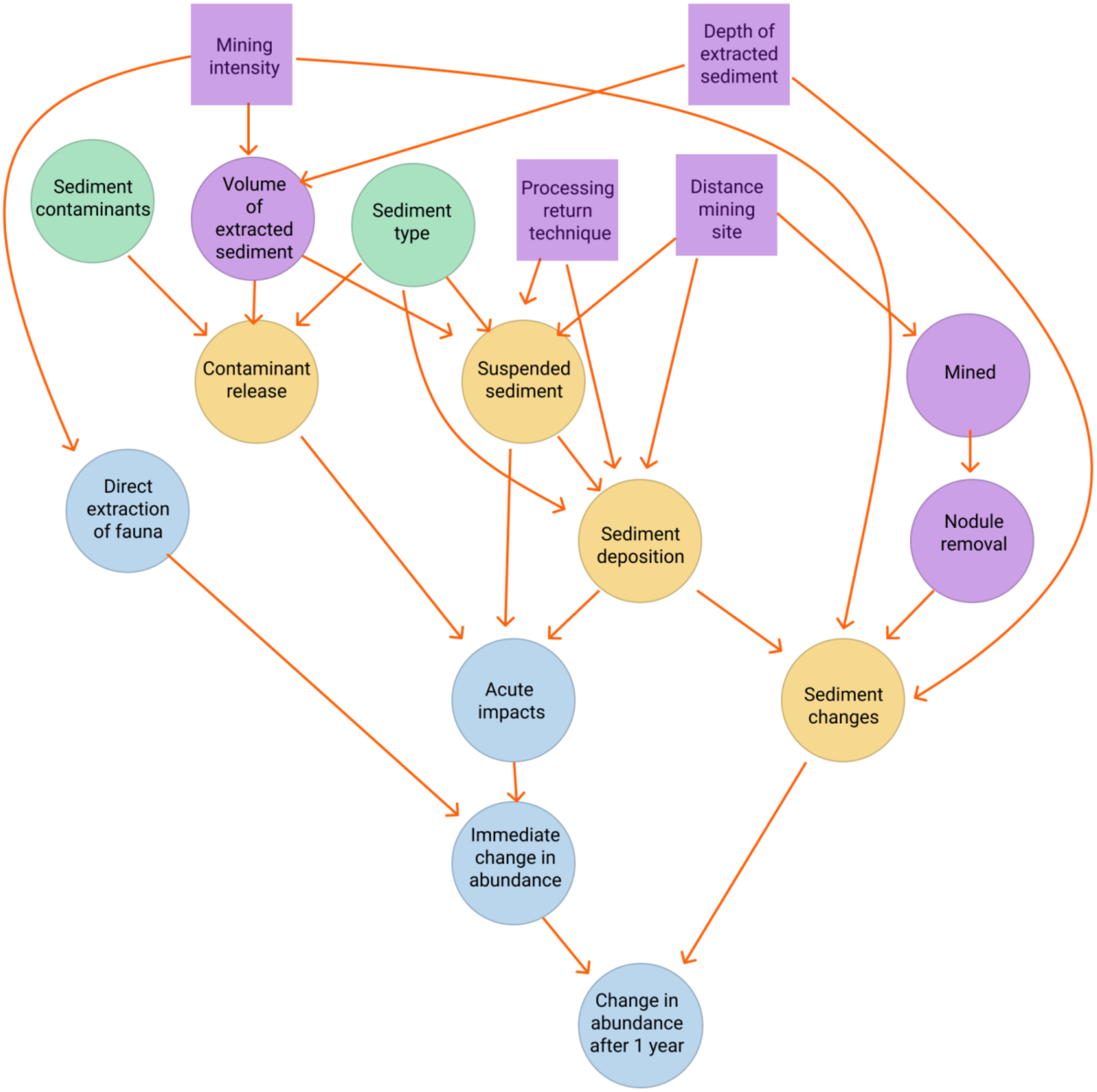

Figure S1. Subset of the Bayesian network structure for one functional group. The arrows (arcs) describe conditional dependencies between variables (variables are conditional to the nodes that connect to them). The network shows how the nodes higher up in the network (variables describing the mining activity and the environmental conditions) flow down to changes in the environment and impacts on benthic fauna. The mining scenario may be controlled by changing the states of the decision nodes (depicted by squares).

The estimates for one year after disturbance were estimated as the relative change in abundance from the pre-disturbed state. The initial change in abundance was considered so that the change in abundance after one year was conditional to the initial loss in abundance and the change in

habitat quality (resulting from seabed disturbance, see Fig. S1). The experts were asked to consider the combined effect of 1) abundance change from the initial disturbance, 2) effect of changes in the sediment environment for the recovery of the organisms (encompassing a variety of changes from which were considered as a summary variable to characterise minor to significant sediment changes, including the loss of phosphorite nodules). A complete recovery after one year would mean that there is no change in abundance after one year, whereas no recovery would mean that the change in abundance is the same as immediately after disturbance.

The affinity to substrate of the different functional groups was considered when making the estimates (e.g., sessile invertebrate megafauna are more sensitive to loss of the hard substrate (including nodules) and were not estimated to be able to recover if the sediment changes were significant). We note that combining the sediment changes into one composite variable is a simplification that was necessary in the scope of this study but is an important aspect to be improved (i.e., considering sediment changes and loss of nodules separately, and more carefully accounting for changes in the sediment environment and its recovery). Similarly, while our model does not consider net increases in abundance of any faunal group, such as scavengers and early colonizers that may benefit from the increase in organic matter (i.e., the relative change in abundance compared to pre-disturbed community cannot be positive, only negative), the impacts of increased food source was considered when making the impact estimates, and for instance some of the mobile benthos was expected to benefit from the food increase and thus recover faster.

While the uncertainties in the estimates are usually thought to be embedded in the probability estimates (i.e., the wider the distribution, the lower the confidence in the estimate is thought to be), we decided to use an additional metric of certainty, because the probability estimates also encompassed the variability in the functional groups consisting of several species with differential responses to disturbance and recovery. This was done by reviewing the information available for each of the model parameters (data, literature, expert opinion) and discussing how confident the experts were about the impact estimates.

It is important to note that with a high number of variable, such as in this study, using an elicitation tool for filling the CPTs is often the only feasible solution when there are many variables to quantify (Pollino et al., 2007). However, the use of a parameter-based estimation method (such as the beta interpolation used in this study), while easy to carry out, may produce estimates that do not fully reflect the expert’s complex views on a topic. To avoid producing incorrect estimates in this BN modelling, all CPTs generated with the interpolation tool were reviewed both by the expert team and the modeller coordinating the effort. When these caveats are considered in the quantification process, the use of interpolation tools provides a useful option for evaluating the effects of multiple stressors, which would otherwise be impossible to quantify due to the high number of possible probability entries required.

Step 5 Application to scenarios

To evaluate the effect of changes in the mining operations on the impacts on benthos, we defined two alternative mining scenarios. These scenarios, which we define as a combination of specific states of the decision variables that describe the overall mining process are assumed to be controlled by the party responsible for the mining operation. We chose to evaluate two scenarios: one characterising the most likely mining operation on the Chatham Rise, and another more similar to surface collectors that may be used for abyssal deep-sea mining. The scenarios were defined by experts based on the description of the planned mining activities on the Chatham Rise (Chatham Rock Phosphate, 2014). All the other variables in the model are further affected by these decision variables (Fig. S1). It should be emphasised that the model can be queried for any combination of variables, and we have presented only a limited number of possible outcomes. The BN model was queried on these two scenarios by setting the values of the decision variables (details included in the R script available at https://github.com/lkaikkonen/CR-ERA).

Step 6 Model evaluation

The model outcomes were evaluated in a series of meetings with the expert group and some of the CPTs were adjusted to better reflect the expected outcomes from the mining disturbance especially regarding the spatial scale of the impacts.

REFERENCES

Aguilera, P. A., Fernández, A., Fernández, R., Rumí, R., & Salmerón, A. (2011). Bayesian networks in environmental modelling. *Environmental Modelling & Software*, *26*(12), 1376–1388.

Barons, M. J., Mascaro, S., & Hanea, A. M. (2022). Balancing the Elicitation Burden and the Richness of Expert Input When Quantifying Discrete Bayesian Networks. *Risk Analysis*, *42*(6), 1196–1234. https://doi.org/10.1111/risa.13772

Chatham Rock Phosphate. (2014). *Proposed Mining Operation, Chatham Rise. Marine Consent Application and Environmental Impact Assessment Report.*

Chen, S. H., & Pollino, C. A. (2012). Good practice in Bayesian network modelling. *ENVIRONMENTAL MODELLING & SOFTWARE*, *37*, 134–145. https://doi.org/10.1016/j.envsoft.2012.03.012

Eigaard, O. R., Bastardie, F., Breen, M., Dinesen, G. E., Hintzen, N. T., Laffargue, P., Mortensen, L. O., Nielsen, J. R., Nilsson, H. C., & O'Neill, F. G. (2016). Estimating

seabed pressure from demersal trawls, seines, and dredges based on gear design and dimensions. *ICES Journal of Marine Science*, *73*(suppl_1), i27–i43.

Frontin-Rollet, G. E. (2017). *The Geochemistry and Ecotoxicity of Offshore New Zealand Phosphorites.* [Thesis, Te Herenga Waka-Victoria University of Wellington.]. https://doi.org/10.26686/wgtn.17072729.v1

Hassall, K. L., Dailey, G., Zawadzka, J., Milne, A. E., Harris, J. A., Corstanje, R., & Whitmore, A. P. (2019). Facilitating the elicitation of beliefs for use in Bayesian Belief modelling. *Environmental Modelling & Software*, *122*, 104539. https://doi.org/10.1016/j.envsoft.2019.104539

Hauton, C., Brown, A., Thatje, S., Mestre, N. C., Bebianno, M. J., Martins, I., Bettencourt, R., Canals, M., Sanchez-Vidal, A., Shillito, B., Ravaux, J., Zbinden, M., Duperron, S., Mevenkamp, L., Vanreusel, A., Gambi, C., Dell'Anno, A., Danovaro, R., Gunn, V., & Weaver, P. (2017). Identifying Toxic Impacts of Metals Potentially Released during Deep-Sea Mining—A Synthesis of the Challenges to Quantifying Risk. *Frontiers in Marine Science*, *4*. https://doi.org/10.3389/fmars.2017.00368

Hewitt, J. E., & Lohrer, A. M. (2013). *Impacts of sedimentation arising from mining on the Chatham Rise. Prepared for Chatham Rock Phosphate Ltd.* NIWA Client Report HAM2012-132.

Hewitt, J. E., Lundquist, C. J., & Ellis, J. (2018). Assessing sensitivities of marine areas to stressors based on biological traits. *Conservation Biology*.

Kaikkonen, L., Helle, I., Kostamo, K., Kuikka, S., Törnroos, A., Nygård, H., Venesjärvi, R., & Uusitalo, L. (2021a). Causal Approach to Determining the Environmental Risks of Seabed Mining. *Environmental Science & Technology*, *55*(13), 8502–8513. https://doi.org/10.1021/acs.est.1c01241

Kaikkonen, L., Parviainen, T., Rahikainen, M., Uusitalo, L., & Lehikoinen, A. (2021b). Bayesian Networks in Environmental Risk Assessment: A Review. *Integrated Environmental Assessment and Management*, *17*(1), 62–78. https://doi.org/10.1002/ieam.4332

Lescinski, J., Jeuken, C., Cronin, K., Vroom, J., & Elias, E. (2014). *Modeling Investigations on Mine Tailing Plume Dispersion on the Chatham Rise1209110*. Technical Report. 2014. Available online: https://www. epa. govt. nz/assets ….

Lundquist, C. J., Bowden, D., Cartner, K., Stephenson, F., Tuck, I., & Hewitt, J. E. (2018). Assessing Benthic Responses to Fishing Disturbance Over Broad Spatial Scales That Incorporate High Environmental Variation. *Frontiers in Marine Science, 5*. https://doi.org/10.3389/fmars.2018.00405

Mobilia, V., Cummings, V. J., Clark, M. R., Tracey, D., & Bell, J. J. (2021). Short-term physiological responses of the New Zealand deep-sea sponge Ecionemia novaezealandiae to elevated concentrations of suspended sediments. *Journal of Experimental Marine Biology and Ecology*, *541*, 151579. https://doi.org/10.1016/j.jembe.2021.151579

Mobilia, V., Tracey, D. M., Cummings, V., Clark, M. R., Woods, L., & Bell, J. (2023). Effects of sediment pulses on the deep-sea coral Goniocorella dumosa. *New Zealand Journal of Marine and Freshwater Research*, *0*(0), 1–17. https://doi.org/10.1080/00288330.2023.2230154

Muñoz-Royo, C., Ouillon, R., El Mousadik, S., Alford, M. H., & Peacock, T. (2022). An in situ study of abyssal turbidity-current sediment plumes generated by a deep seabed polymetallic nodule mining preprototype collector vehicle. *Science Advances*, *8*(38), eabn1219. https://doi.org/10.1126/sciadv.abn1219

Table S3. Experts involved in the expert elicitation for providing probability estimates and/or supporting information for the model parameterisation. The specific fields of expertise are not comprehensive.

**Name**	**Expertise**	**Model variables contributed to**	**Institute**
Ashley Rowden	Marine Ecology	Infauna	National Institute of Water and Atmospheric Research and Victoria University
Daniel Leduc	Marine Ecology	Infauna	National Institute of Water and Atmospheric Research
David Bowden	Marine Ecology	Mobile epibenthos and hyperbenthos	National Institute of Water and Atmospheric Research
Vonda Commings	Marine Ecology	Sessile megafauna	National Institute of Water and Atmospheric Research
Jennifer Beaumont	Marine Ecology	Sessile megafauna	National Institute of Water and Atmospheric Research
Di Tracey	Marine Ecology	Sessile megafauna	National Institute of Water and Atmospheric Research
Savannah Goode	Marine Ecology	Sessile megafauna	National Institute of Water and Atmospheric Research and Victoria University
Scott Nodder	Marine geology and biogeochemistry	Sediment changes, suspended sediment, sediment deposition	National Institute of Water and Atmospheric Research

Table S4. Details of variable discretisation and parameterisation

Variable name	Description	Variable states	Discretisation method	Parameterisation method
Depth of extracted sediment	Depth of sediment extracted by the mining tool	<10cm / 10-30 cm / >30cm	Variable states were defined by experts based on literature to describe different potential seabed mining operations and to be applicable to other types of seabed disturbance, such a bottom trawling. The lowest class describes surface collector operations or low-penetration bottom trawling operations, 10-30cm traditional bottom trawling (Eigaard et al., 2016; Muñoz-Royo et al., 2022), and the >30cm the most likely extraction depth for the planned phosphorite mining (Chatham Rock Phosphate, 2014).	Not applicable
Processing return technique	Depth of processing return water and sediment	10m from seafloor / at the seafloor	Discrete classes were retrieved from the Chatham Rock Phosphate mining consent application (Chatham Rock Phosphate, 2014).	Not applicable
Mining intensity	Proportion of area mined (or disturbed in a non-mining application) within a discrete mining block	50% / 75% / 100%	Three discrete classes were decided by experts to show potential variation in the mining intensity and to align with description given in the Chatham Rock Phosphate Marine Consent Application (Chatham Rock Phosphate, 2014).	Not applicable (decision variable)
Distance from the mining block	Distance from the mining block	Inside mining block / Near-field / Far-field	The impacted area was split into three zones based on expert opinion and available results in the Chatham Rock Phosphate Marine Consent Application (Chatham Rock Phosphate, 2014).	Not applicable (decision variable)
Volume of extracted sediment	Volume of sediment removed by a mining operation tool (as	Low-medium-high	Volume of sediment was not given a numerical value due to the conceptual nature of the model and lack of exact information on the volumes to be	Literature, expert assessment

Variable name	Description	Variable states	Discretisation method	Parameterisation method
	the initial removal)		extracted in literature. The three relative states were chosen by experts using available information in the Chatham Rock Phosphate Marine Consent Application (Chatham Rock Phosphate, 2014). Note that the low-med-high will be relative to the type of seabed operation to be evaluated with the model (mining, trawling etc.)	
Particle size composition of the extracted sediment	Proportion of fine and coarser particles of the extracted substrate and in the composite sediment plume	Mostly silt and clay (fine) / mix of fine and coarse particles / mostly coarse (sand and gravel)	Field data from the area	Field survey particle size data before and after disturbance was used to inform probability distribution.
Sediment contaminants	Concentration of potentially harmful substances in the sediment and the mineral material to be extracted	Low-Medium-High	Three relative classes decide by experts due to high number of potential biologically harmful contaminants and lack of information on their presence (Frontin-Rollet, 2017; Hauton et al., 2017).	Probability estimates provided by experts using literature as supporting material
Nodule removal	Proportion of phosphorite nodules removed from a discrete mining block.	Yes / No	Not applicable	Not applicable (decision variable)
Suspended sediment	Total suspended solids concentration near the seafloor resulting both from the processing return	Low: <10 mg L^{-1} / Moderate: 11-50 mg L^{-1} / High:>100 mg L^{-1}	Three discrete classes were chosen to align with the SSC levels used in the lab experiments, levels detected in the disturbance experiment and baseline studies, and modelled SSC values from the sediment plume modelling dispersal for Chatham Rock Phosphate	Conditional probabilities were estimated by experts using the field data measurements and modelled values on the SSC dispersal as supporting

Variable name	Description	Variable states	Discretisation method	Parameterisation method
	and mining tool operation		(Lescinski et al., 2014) to deliver a contrast in biological responses in terms of extraction impacts.	information. (Lescinski et al., 2014).
Sediment deposition	Depth of sediment deposited from the composite suspended sediment plume	Low: <1-2 cm / Moderate: 2-5 cm / High:10-25cm	Natural sedimentation rates estimated from Lander data picking up SCIP disturbance compared with mooring data (for natural sedimentation rates) gave inform this. Likely deposition rates from mining operations and relevant thresholds for benthic fauna from (Chatham Rock Phosphate, 2014; Hewitt & Lohrer, 2013)	Field measurements and expert assessment
Contaminant release	Release of contaminants from the sediment plume to the seabed water column	Nonsignificant / Significant release of contaminants	Due to scarce evidence on the release of contaminants and their effect on benthic fauna, a binary variable was deemed most appropriate.	Probability was based on expert assessment, only a marginal probability was given for significant response due to lack of evidence.
Changes in sediment characteristics	Measure of changes in the sediment environment affecting habitat quality for benthic organisms, including changes in: • Grain size • Sediment porosity	Minor to no changes / Significant changes	Due to scarce evidence on the release of contaminants and their effect on benthic fauna, a binary variable was deemed most appropriate.	Field measurements data on sediment particle size change was used to calculate relative change in mud content. Total sediment change was estimated as the combination of nodule removal and sediment particle change and final probabilities were given by experts based on the field data.

Variable name	Description	Variable states	Discretisation method	Parameterisation method
	• Sediment compaction<br>• Organic enrichment			
All benthic fauna	Relative change in abundance of benthic fauna		Five discrete classes decided by expert team to divide the continuous abundance change to provide a comparison and to limit the number of required probability entries to facilitate probability elicitation	Field measurements, used to inform the probability estimation. Probability estimation done with experts using and reviewed with the entire expert groups to ensure consistency between estimates for different faunal groups.
