# Supplementary material for: Probabilistic ecological risk assessment for deep‐sea mining: A Bayesian network for Chatham Rise, Pacific Ocean
Source: Ecol Appl. 2024 Nov 25;35(1):e3064. doi: 10.1002/eap.3064 (PMC11734116; doi:10.1002/eap.3064)
Supplement: Supplementary file 2 — Appendix S2. [file EAP-35-e3064-s001.pdf]

Probabilistic ecological risk assessment for deep-sea mining: A Bayesian network for Chatham Rise, Pacific Ocean

Laura Kaikkonen, Malcolm R. Clark, Daniel Leduc, Scott D. Nodder, Ashley A. Rowden, David A. Bowden, Jennifer Beaumont, Vonda Cummings

Appendix S2

- Appendix S2 Figures S1-S6: Full results of model runs under different scenarios for all evaluated benthic functional groups

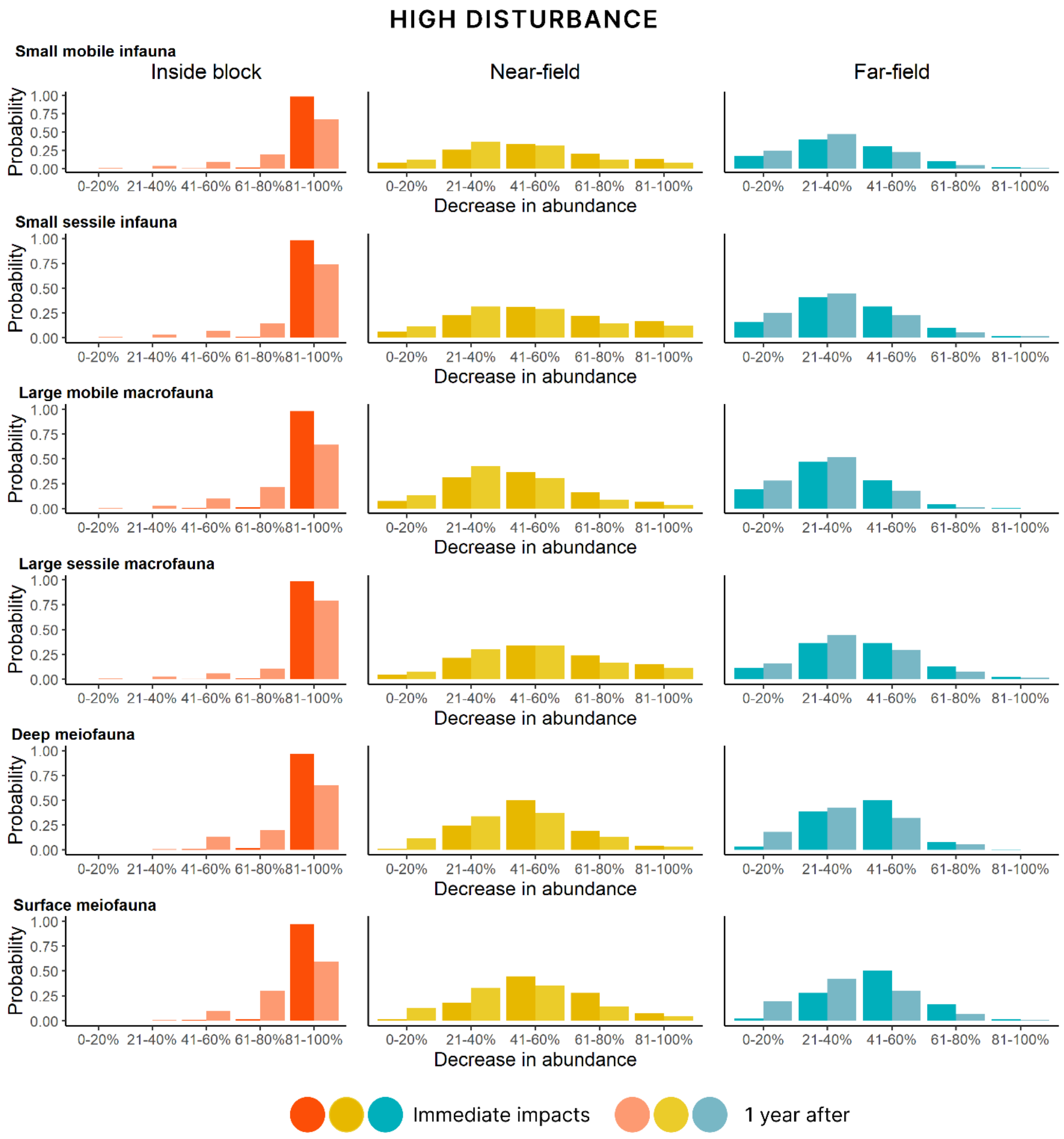

Figure S1. Impacts on infauna inside the mining block (left panel), in the near-field area directly adjacent to the mined area (middle), and outside the mining block in the far-field (right panel) under the high disturbance scenario. Immediate impacts are noted in a dark shade and impacts after one year in a lighter shade.

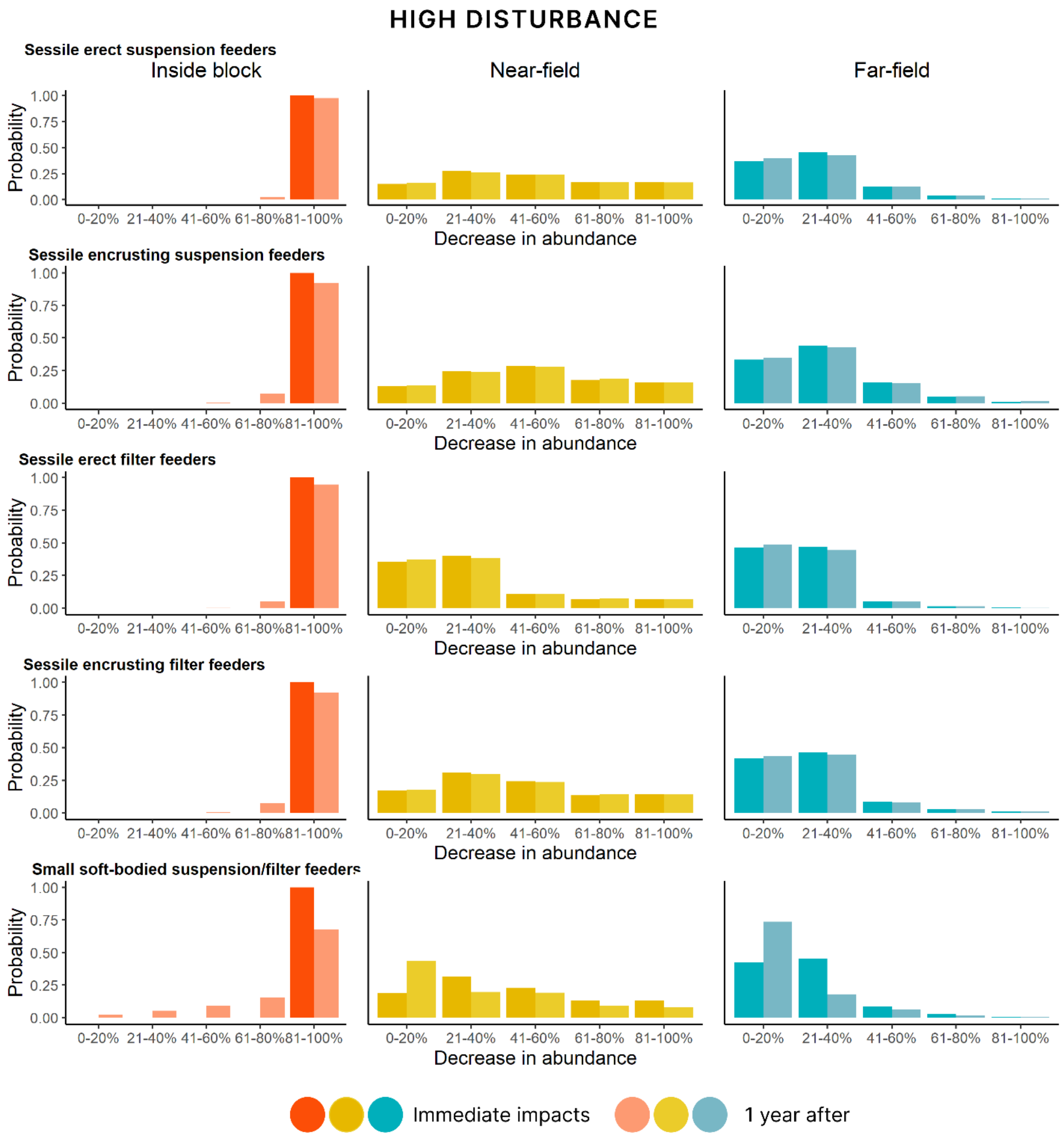

Figure S2. Impacts on sessile mega-epibenthos inside the mining block (left panel), in the near-field area directly adjacent to the mined area (middle), and outside the mining block in the far-field (right panel) under the high disturbance scenario. Immediate impacts are noted in a dark shade and impacts after one year in a lighter shade.

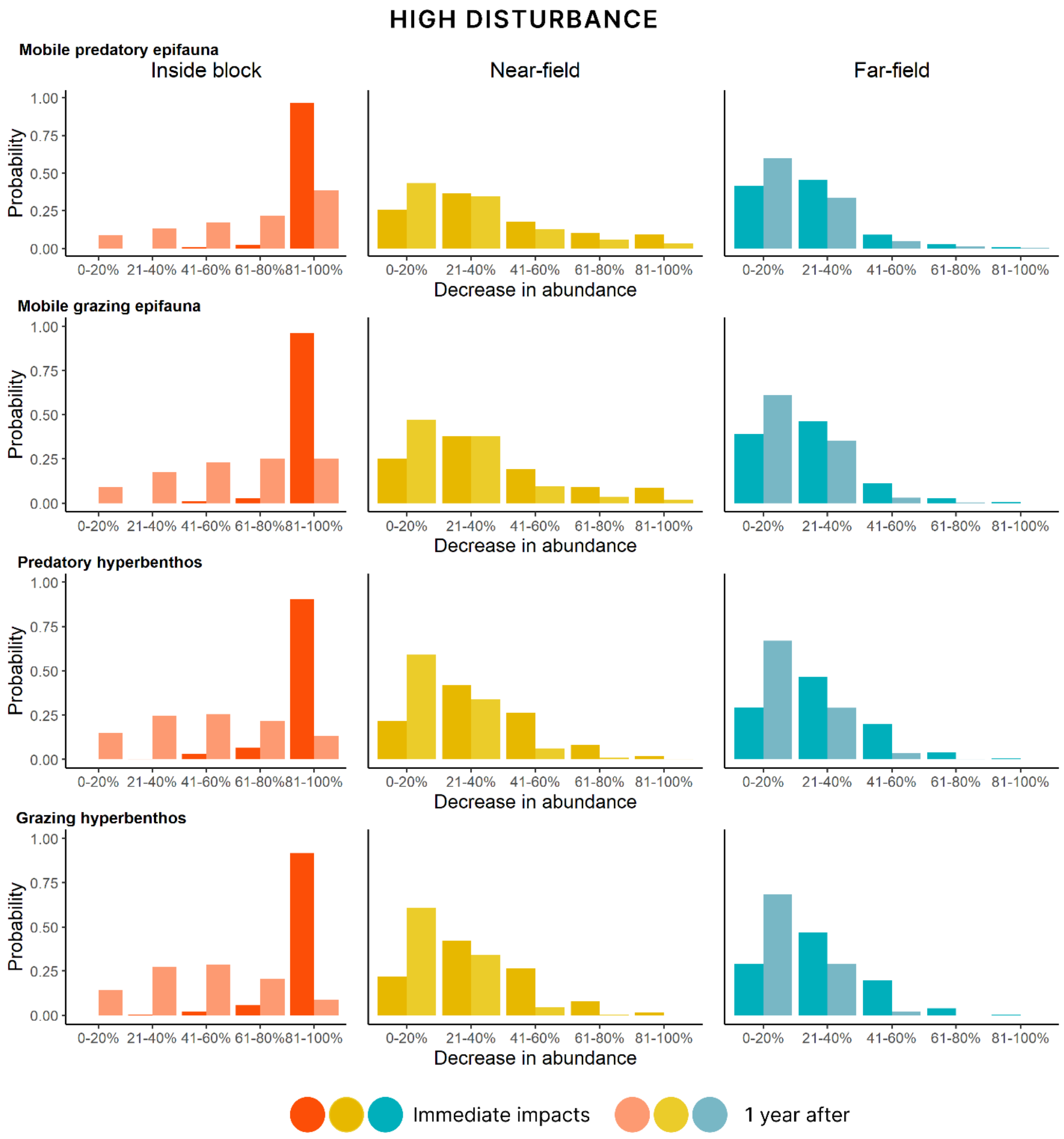

Figure S3. Impacts on mobile epibenthos inside the mining block (left panel), in the near-field area directly adjacent to the mined area (middle), and outside the mining block in the far-field (right panel) under the high disturbance scenario. Immediate impacts are noted in a dark shade and impacts after one year in a lighter shade.

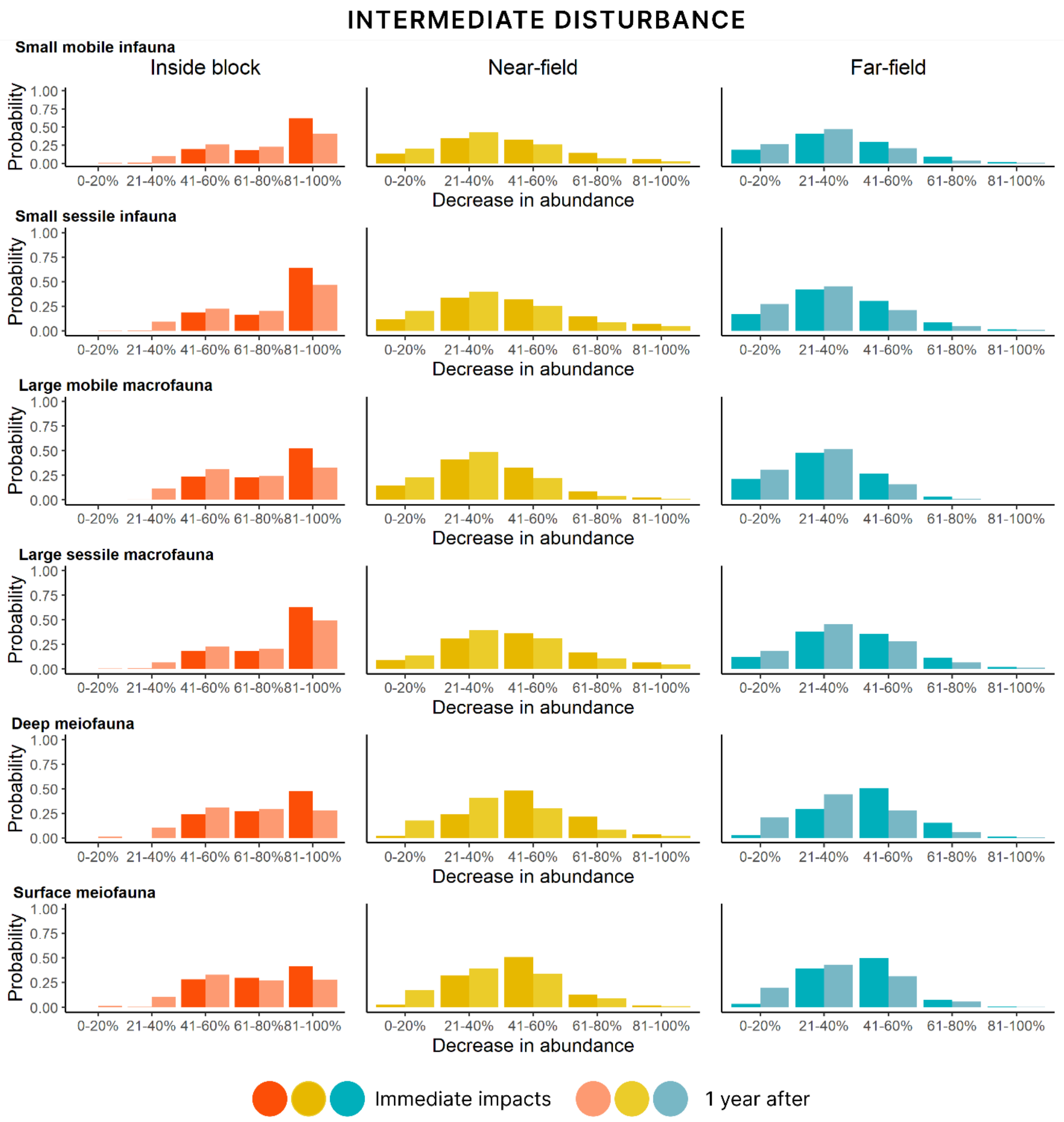

Figure S4. Impacts on infauna inside the mining block (left panel), in the near-field area directly adjacent to the mined area (middle), and outside the mining block in the far-field (right panel) under the Intermediate disturbance scenario. Immediate impacts are noted in a dark shade and impacts after one year in a lighter shade.

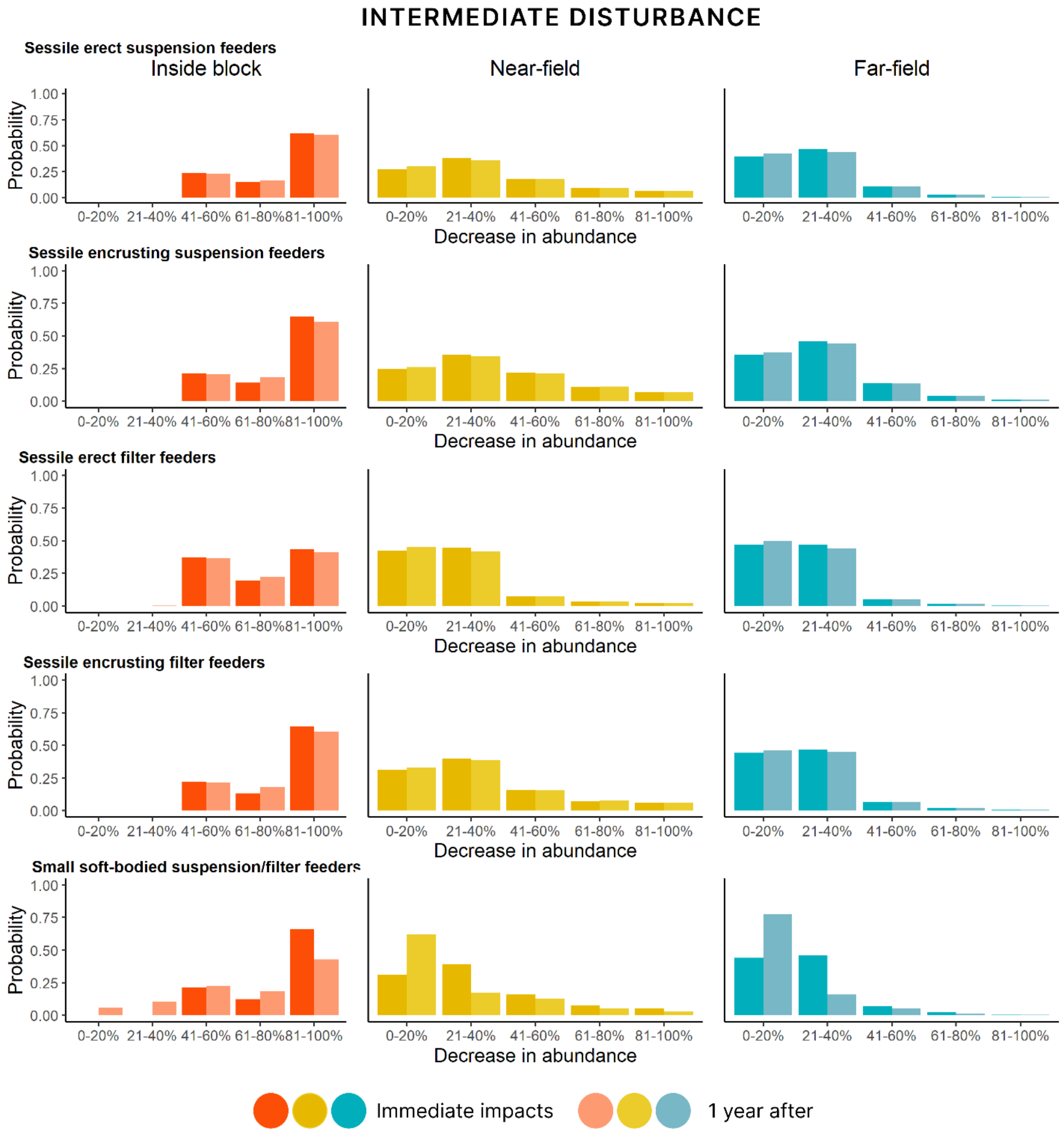

Figure S5. Impacts on sessile fauna inside the mining block (left panel), in the near-field area directly adjacent to the mined area (middle), and outside the mining block in the far-field (right panel) under the Intermediate disturbance scenario. Immediate impacts are noted in a dark shade and impacts after one year in a lighter shade.

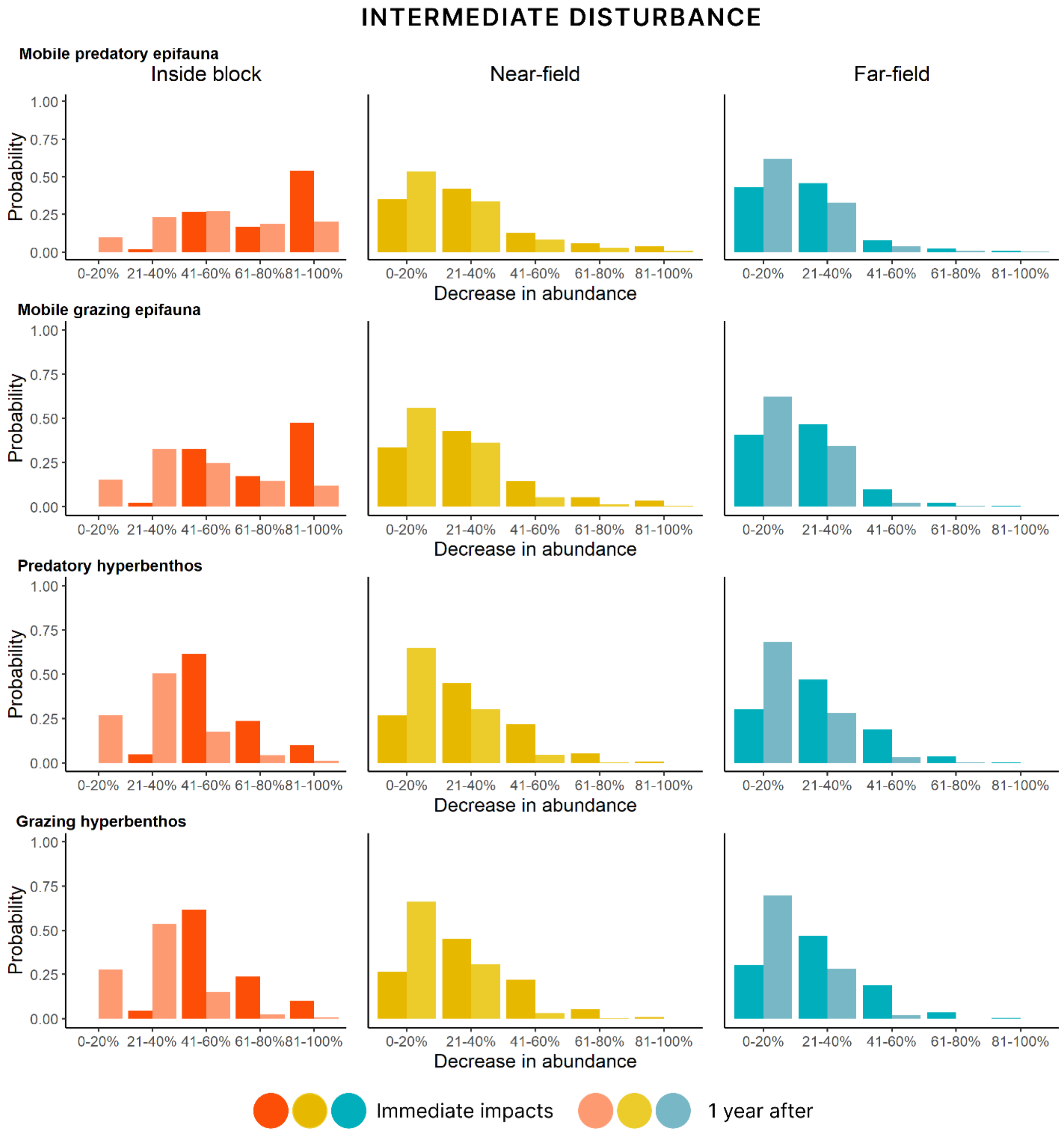

Figure S6. Impacts on mobile fauna inside the mining block (left panel), in the near-field area directly adjacent to the mined area (middle), and outside the mining block in the far-field (right panel) under the Intermediate disturbance scenario. Immediate impacts are noted in a dark shade and impacts after one year in a lighter shade.
